# Presence does not imply activity: DNA and RNA patterns differ in response to salt perturbation in anaerobic digestion

**DOI:** 10.1186/s13068-016-0652-5

**Published:** 2016-11-09

**Authors:** Jo De Vrieze, Leticia Regueiro, Ruben Props, Ramiro Vilchez-Vargas, Ruy Jáuregui, Dietmar H. Pieper, Juan M. Lema, Marta Carballa

**Affiliations:** 1 Center for Microbial Ecology and Technology (CMET), Ghent University, Coupure Links 653, B-9000 Ghent, Belgium; 2 Department of Chemical Engineering, School of Engineering, University of Santiago de Compostela, Rúa Lope Gomez de Marzoa s/n, E-15782 Santiago de Compostela, Spain; 3 Microbial Interactions and Processes Research Group, Helmholtz Centre for Infection Research (HZI), Brunswick, Germany; 4 AgResearch, Tennent Drive, Palmerston North, 4442 New Zealand

**Keywords:** Archaea, Biogas, Illumina sequencing, Methanogenesis, Salinity

## Abstract

**Background:**

The microbial community in anaerobic digestion is mainly monitored by means of DNA-based methods. This may lead to incorrect interpretation of the community parameters, because microbial abundance does not necessarily reflect activity. In this research, the difference between microbial community response on DNA (total community) and RNA (active community) based on the 16S rRNA (gene) with respect to salt concentration and response time was evaluated.

**Results:**

The application of higher NaCl concentrations resulted in a decrease in methane production. A stronger and faster response to salt concentration was observed on RNA level. This was reflected in terms of microbial community composition and organization, as richness, evenness, and overall diversity were differentially impacted. A higher divergence of community structure was observed on RNA level as well, indicating that total community composition depends on deterministic processes, while the active community is determined by stochastic processes. *Methanosaeta* was identified as the most abundant methanogen on DNA level, but its relative abundance decreased on RNA level, related to salt perturbation.

**Conclusions:**

This research demonstrated the need for RNA-based community screening to obtain reliable information on actual community parameters and to identify key species that determine process stability.

**Electronic supplementary material:**

The online version of this article (doi:10.1186/s13068-016-0652-5) contains supplementary material, which is available to authorized users.

## Background

Anaerobic digestion (AD) contains a complex microbial community that produces biogas from organic waste streams. The four different steps in the AD process are carried out by distinct groups of micro-organisms, each requiring specific conditions to ensure optimal performance [1]. Operational control is of crucial importance to ensure maximal biogas production rates [2]. At present, the stability of the AD process is mainly monitored based on the conventional parameters, such as pH, volatile fatty acid (VFA) concentrations, alkalinity, and biogas composition [3–5]. More complex stability indicators, such as the Ripley index [6] and the VFA:Ca ratio [7], were developed to estimate AD stability. None of these parameters consider microbial community dynamics or abrupt changes, and, therefore, cannot be used for long-term stability prediction.

Monitoring of the microbial community could lead to a better stability prediction and even prevent operational failure [8, 9]. The development and subsequent application of culture-independent molecular techniques, such as 16S rRNA gene amplicon sequencing, have led to a strong increase in knowledge concerning the microbial community in the AD process [10, 11]. Microbial community evenness [12–14] and dynamics [8, 15, 16], whether or not influenced by a suitable inoculum [17], reflect the microbial community structure and organization, and can strongly impact AD process resilience. The identification of the main microbial groups, performing specific reactions, and their role in the AD process has paved the road for the development of microbial community-based process stability indicators [18–23].

The majority of studies performing amplicon sequencing focused on the 16S rRNA gene, which resulted in relative abundance profiles of the various microbial groups potentially important for AD process performance [10, 11]. This approach provides only limited information on the active microbial community, and consequently, on their potential involvement in the process [10]. An approach that takes the 16S rRNA itself into consideration could lead to a more accurate estimation of the active microbial community. In the few studies performing RNA-, protein-, or metabolite-based community analysis, it was shown that methanogens had a higher level of activity in comparison with their absolute or relative abundance [24–26]. The application of RNA-, protein-, and/or metabolite-based methods would enable a better understanding of the influence of operational parameters on the microbial community performance [27]. The response time on RNA, protein, or metabolite level to changes in operational parameters is much faster than on DNA level, especially for the slow growing methanogens [28]. This allows the possibility for a more accurate measurement of the microbial community structure and activity dynamics, which could entail a more specific adjustment of selective operational parameters, such as pH or organic loading rate [29, 30].

In this research, the effect of an increased salinity on total and active microbial community composition was evaluated. The actual effects of salt concentration in function of time were assessed on the level of the 16S rRNA gene (DNA) and the 16S rRNA itself, to validate their difference in response. The RNA level is considered a *proxy* of the active microbial community, while the DNA level represents the total microbial community. The potentially strong difference in 16S rRNA gene copy number between micro-organisms has to be considered [31], which is why in this study a major focus was placed on the RNA/DNA ratio of the different micro-organisms, as well as its changes. The 16S rRNA (gene)-based species counting can be considered very useful to estimate broad and strong changes over time, yet a metagenomics approach will be needed to identify subtler changes [32, 33]. Hence, the focus of this study was mainly on strong changes of the microbial community on DNA and RNA levels, and shifts in the RNA/DNA ratio. It was hypothesized that (1) a higher degree of change in the microbial community and faster response should be observed on RNA level, compared with DNA, and (2) the methanogens, due to their greater sensitivity to high salt concentrations [34, 35], would show a higher degree of change than the bacterial community, both on DNA and RNA levels.

## Methods

### Substrates and inoculum

A mixture of primary sludge and waste activated sludge in a 70:30 weight ratio was used as substrate to feed the reactors (Additional file 1: Table S1). Both sludge types were collected from the municipal wastewater treatment plant of Santiago de Compostela, and the anaerobic inoculum sludge sample originated from the full-scale mesophilic sludge digester (Additional file 1: Table S2).

### Experimental design

#### Mother reactors

Three identical lab-scale continuous stirred tank reactors with a working volume of 2 L were operated for a period of 42 days at mesophilic temperature (37 °C). Stirring took place on a shaker at 120 rpm. Each reactor was connected to a Ritter milligas counter (Dr. Ing. Ritter Apparatebau GmbH, Bochum, Germany) to monitor biogas production. A sludge retention time of 15 days was applied during the first 7 days of the experiment after which it was increased to 30 days for the entire remaining period. The organic loading rate was fixed at 1 g COD L^−1^ d^−1^ (chemical oxygen demand) to avoid overloading. The inoculum was diluted with tap water to a volatile solids concentration of 15 g L^−1^. Feeding of the reactors was performed three times a week, and fresh feed was prepared for every feeding. Biogas production and composition were determined three times a week, and reported at standard temperature and pressure (STP, 273.15 K, and 101,325 Pa) conditions. The pH was also measured three times a week before feeding, while alkalinity, Ripley index, and VFA concentration were determined on weekly basis.

#### Short-term perturbation test

The short-term perturbation test consisted of four different treatments, each carried out in triplicate in identical lab-scale continuous stirred tank reactors with a total volume of 500 mL and a working volume of 400 mL. First, each reactor was inoculated with 400 mL of anaerobic sludge from the mixture of the three mother reactors (Additional file 1: Table S3). Second, a single pulse of the primary and waste activated sludge mixture was added to obtain a single substrate load of 5.0 g COD L^−1^. Finally, a single pulse of NaCl was added, resulting in a surplus concentration of 0 (control treatment), 5, 10, and 20 g Na^+^ L^−1^, respectively, and the reactors were sealed to maintain anaerobic conditions. The twelve reactors were operated at mesophilic conditions (37 °C) on a shaker (120 rpm) for a period of 14 days. Biogas production and composition were measured on a daily basis by means of pressure build-up, using an in-house constructed pressure transducer, and reported at STP conditions. Samples for pH, VFA, and cation analysis were taken at the end of the test. Samples for DNA and RNA analysis were taken on day 0, 1, 2, 4, 7, and 14 (T0–T5), and stored at −80 °C prior to analysis.

### Microbial community analysis

#### DNA and RNA extraction, amplicon sequencing, and amplicon sequence processing

The extraction of genomic DNA and total RNA was carried out using the PowerSoil^®^ DNA Isolation kit and RNA PowerSoil^®^ Total RNA Isolation Kit (MoBio Laboratories, Inc., Carlsbad, CA, USA), respectively, according to manufacturer’s instructions after which the Illustra Ready-To-Go RT-PCR Beads (GE Healthcare, Buckinghamshire, UK) were used for conversion of the RNA to cDNA. The quantity and quality of the resulting DNA and cDNA were determined with a Nanodrop 2000c (Thermo Scientific, Wilmington, DE, USA). Microbial community analysis was carried out by means of high-throughput amplicon sequencing using 250 bp paired-end sequencing chemistry (MiSeq Illumina), both on the DNA and cDNA samples. The general primers 807F and 1050R, targeting the V5–V6 region of the 16S rRNA gene of both bacteria and archaea [36], were used as previously described [37]. The preparation of libraries for barcode sequencing and data-set quality filtering were carried out as described by Camarinha-Silva, et al. [38]. A quality filter that runs a sliding window of 10% of the read length at a time, and calculates the local average score based on the Phred quality score of the fastq file, was used to trim the 3′-ends of the reads that fall below a quality score of 10. All reads that had ambiguous bases, any mismatches within the primers and barcodes, or more than ten homopolymer stretches were discarded. Primers and barcodes were then trimmed from each read. Reads were trimmed conservatively to 140 nucleotides, and the paired ends were subsequently matched to give 280 nt. The Mothur command *unique.seqs* was used to extract the unique reads. The paired-end reads were merged to fully cover the V5–V6 region, and no mismatch was allowed in the overlapping of the forward and reverse reads (Additional file 2). All reads were clustered allowing two mismatches (>99% sequence identity). Chimera detection was performed by UCHIME in de novo mode, using the sequence abundances of combined samples as suggested by the developers [39]. UCHIME identified 0.82% of total reads (139 phylotypes) as chimeras. As a significant amount of these reads were sequences documented as originating from type strains (9 phylotypes), isolates (13 additional phylotypes) had been documented in multiple long-sized 16S rRNA gene sequences (23 additional phylotypes) or at least in a single long-sized 16S rRNA gene sequence (11 additional sequences) [40], chimera removal was not performed. The amplicon sequence fragments were assigned to phylotypes, using the Ribosomal Database Project database, based on the Naive Bayesian classification with a confidence threshold of 80% [41].

#### Data processing and statistical analysis

The resulting data set containing the relative abundance of each phylotype in all samples was analysed using the R software, version 3.2.3. (http://www.r-project.org) [42]. Rarefaction curves were generated for each sample to evaluate sampling depth [43, 44], using the *phyloseq* [45] and *vegan* packages [46]. The *vegan* package was also used to determine the diversity indices, and to calculate Bray–Curtis dissimilarity matrices (*vegdist* function). A table containing all samples with the abundance of different phylotypes and their taxonomic assignments was created (Additional file 2), which was used to generated the heat maps on different phylogenetic levels by means of the *pheatmap* package. Reproducibility analysis of the replicates, significant differences in diversity parameters, and variation between Bray–Curtis dissimilarity matrices were determined by means of analysis of variance (ANOVA). Permutational multivariate analysis of variance (PERMANOVA) of the Bray–Curtis dissimilarity matrices was carried out with the vegan package (*adonis* functions). For multivariate abundance analysis, all analyses were conducted with the *mvabund* package and seed 777 [47]. Samples were pruned from phylotypes with a maximum relative abundance lower than 0.1% or that were absent in one of the samples. This was done to focus specifically on the abundant micro-organisms with clear temporal dynamics. After this preprocessing, a forward-selection-based modelling approach was used for testing the relationship between environmental parameters and phylotypes abundances. The mean–variance relationship was modelled by a negative binomial distribution. Before hypothesis testing, all models were verified for accordance with the model assumptions (Additional file 1: Figure S1). Hypothesis testing was performed using likelihood ratio tests with pit resampling (5000 runs). The final model consisted of salt concentration and time as continuous predictors and the reactor replicate and nucleic acid type as categorical predictors. Inference on the model parameters of individual species was assessed using the adjusted *P-*values, calculated after 5000 resampling runs, which accounted for inter-variable correlations.

### Analytical methods

The pH was measured with a Crison 506 standard pH meter (Crison, Barcelona, Spain), equipped with an Ingold U 455 electrode (Mettler-Toledo International Inc., Barcelona, Spain). Total solids (TS), volatile solids (VS), total alkalinity, partial alkalinity, and total ammonia nitrogen (TAN) were determined according to the standard methods [48]. The free ammonia concentration was calculated based on the pH, temperature, and TAN concentration [49]. The Ripley index was defined as the ratio between the intermediate alkalinity (total alkalinity minus partial alkalinity), and total alkalinity [6]. Sodium, potassium, calcium, and magnesium concentrations were measured by means of ion chromatography (IC), using a Metrohm 861 Advanced Compact IC, equipped with a column Metrosep A Supp 5–250 and a 853 CO_2_ Suppressor.

The volatile fatty acids (VFA) were extracted from 2 mL samples using diethyl ether (2 mL), and analysed by means of a gas chromatograph (HP, 5890A) with a glass column (3 m × 2 mm), filled with Chromosor WAW (mesh 100/120), and impregnated with Neopentylglycoladipate (25%) and H_3_PO_4_ (2%), a flame ionization detector and an automatic injector (HP, 7673A). The column, injector, and detector temperatures were set at 105, 260, and 280 °C, respectively. The carrier gas was nitrogen gas, saturated with formic acid, at a flow rate of 24 mL min^−1^. Dry air and hydrogen gas were used as auxiliary gases, with flow rates of 400 and 30 mL min^−1^, respectively. Pivalic acid was used as internal standard. Calibration of the gas chromatograph was carried out using the Volatile Free Acid Mix (Sigma-Aldrich BVBA, Diegem, Belgium), and values were corrected for the partition coefficients of the different VFA in diethyl ether. The lower detection limit was between 10 and 20 mg L^−1^.

Biogas composition was determined by gas chromatography (HP, 5890 Series II), equipped with a thermal conductivity detector. The stainless steel column has a total length of 2 m and an internal diameter of 3.175 mm, and was filled with Poropack Q (mesh 80/100). The temperatures of the injector, column and detector were set at 110, 35, and 110 °C, respectively. Helium was used as carrier gas at a flow of 15 mL min^−1^.

## Results

### Mother reactors performance

Three identical mother reactors were operated for a period of 42 days to obtain a stable microbial community, adapted to the feed sludge mixture. Methane production slowly increased to a final value of 173 ± 27 mL CH_4_ L^−1^ d^−1^ on day 42 (Additional file 1: Figure S2a), which corresponded with a COD conversion efficiency of 49.5 ± 7.8%. The pH remained between 6.91 and 7.22 over the entire period (Additional file 1: Figure S2b), and total VFA decreased from 1561 ± 202 mg COD L^−1^ to values below the limit of detection at the end of the experiment (Additional file 1: Figure S2c). The Ripley index also remained around the threshold value of 0.3 [6] throughout the experiment (Additional file 1: Figure S2d).

### Short-term operational response to salt perturbation

**Fig. 1:**
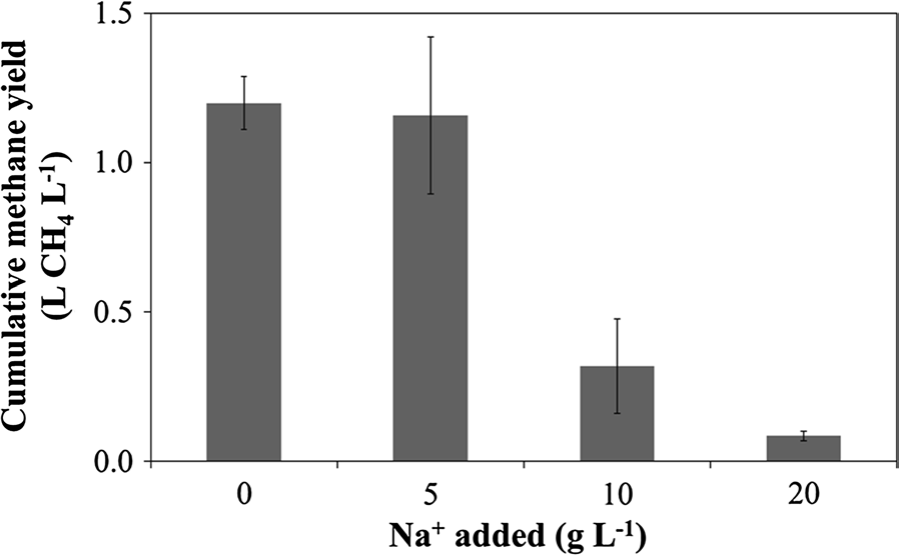
Cumulative methane yield in the short-term perturbation tests after 14 days of operation. Average values of the triplicate reactors are presented, and *error bars* show standard deviations

The triplicate reactors were followed for a period of 14 days after which methane production reached a plateau in each treatment (Additional file 1: Figure S3). Total methane yield reached similar values of 1.20 ± 0.08 and 1.16 ± 0.23 L CH_4_ L^−1^ in the control (no Na^+^ addition) and treatment with 5 g Na^+^ L^−1^, respectively (Fig. 1). This corresponds with a COD substrate conversion to CH_4_ of 68.5 ± 4.5 and 66.1 ± 13.1%, respectively. In contrast, the treatments with 10 and 20 g Na^+^ L^−1^ clearly had a much lower methane production, with values of 0.32 ± 0.14 and 0.08 ± 0.01 L CH_4_ L^−1^, respectively. These values corresponded with a COD conversion to CH_4_ of 18.2 ± 7.9 and 4.9 ± 0.8%, indicating severe inhibition of methanogenesis. These results were confirmed by the increase in VFA concentration to 1.24 ± 0.33 and 1.12 ± 0.46 g COD L^−1^ in the reactors with 10 and 20 g Na^+^ L^−1^, respectively, while no VFA were detected in the control and the treatment with 5 g Na^+^ L^−1^ (Additional file 1: Figure S4a). The accumulation of VFA in the reactors with 10 and 20 g Na^+^ L^−1^ corresponded with 28.6 ± 1.2 and 27.8 ± 0.5%, respectively, of the substrate COD. When adding, however, both COD converted to CH_4_ and VFA together, it seems that both methanogenesis and acidogenesis/acetogenesis were affected in the reactors with 10 and 20 g Na^+^ L^−1^, as the residual VFA concentrations did not compensate for the decrease in methane production. The major fraction of the VFA was acetate, with values of 60.8 ± 9.0% for the reactor with 10 g Na^+^ L^−1^ and 50.2 ± 1.0% for the reactor with 20 g Na^+^ L^−1^. The pH showed a clear decrease in the reactors with 10 and 20 g Na^+^ L^−1^, compared with the initial pH, while this was not the case in the other two reactors (Additional file 1: Figure S4b). The actual Na^+^ concentrations were confirmed by the IC analysis at the end of the experiment, and this confirmed that the Na^+^ concentration in the control treatment was lower than 0.25 g Na^+^ L^−1^ (Additional file 1: Figure S4c).

### Microbial community analysis

Amplicon sequencing analysis resulted in an average of 45,950 ± 19,632 reads per sample, which were clustered into 2787 phylotypes. Complete coverage of the microbial community was confirmed by the rarefaction curves (Additional file 1: Figure S5). Taxonomic resolution allowed to annotate the phylotypes in 31 different phyla, 64 classes, 91 orders, 152 families, and 330 genera. The feed sludge sample was only included in the heat map analysis.

#### Total and active microbial community composition at different phylogenetic levels

**Fig. 2:**
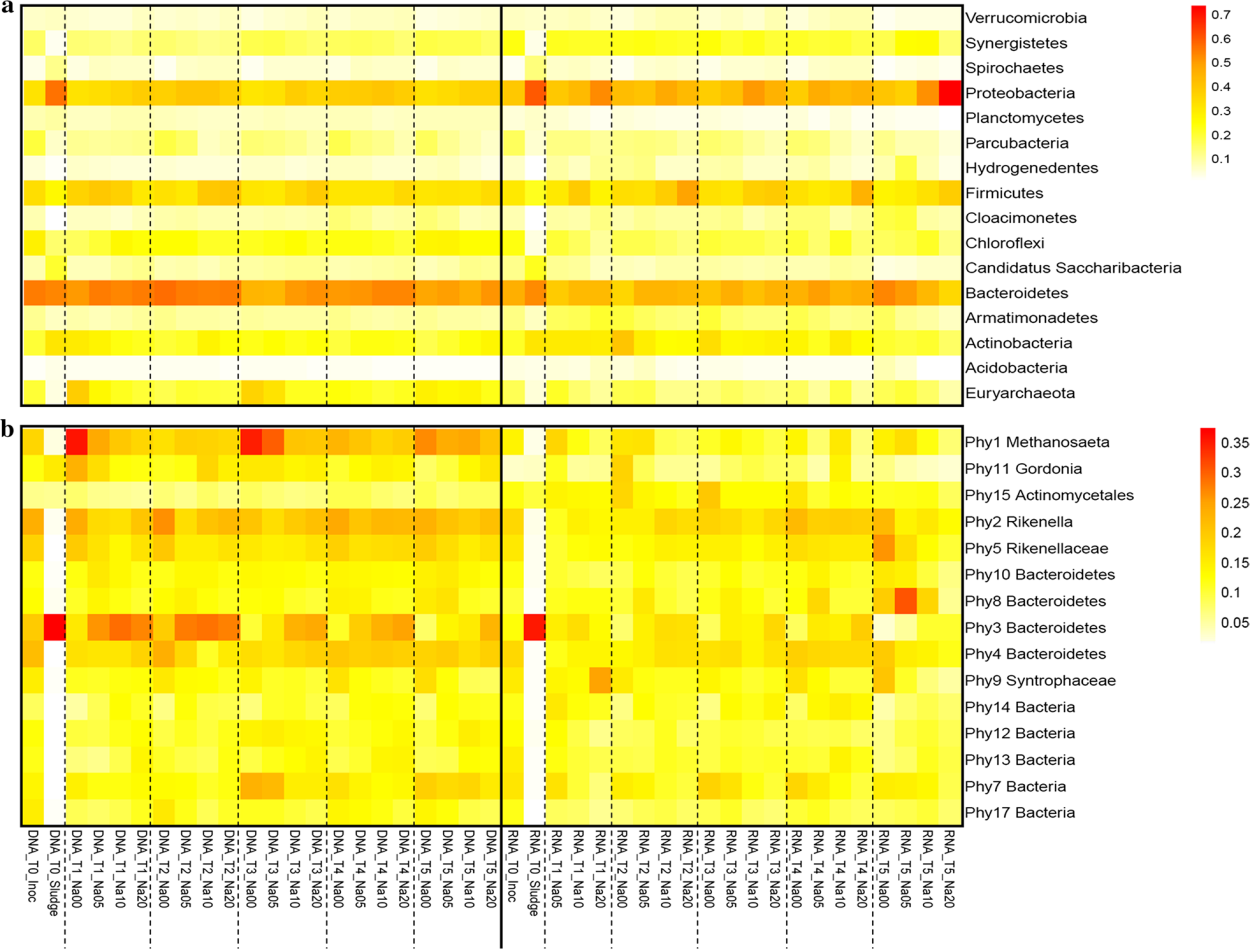
Heat map representing the microbial community of all samples **a** on phylum level at a relative abundance >0.1% averaged over all samples, and **b** the 15 main phylotypes that are present at a relative abundance >1% averaged over all samples. The colour scale ranges from 0 to 70% and 0 to 35% on phylum and phylotype levels, respectively. The different samples were labelled according to the time point (T0–T5) and salt concentration (0, 5, 10 and 20 g Na^+^ L^−1^). The inoculum sample (Inoc) at the start of the experiment as well as the feed sludge (Sludge) were also included in the heat map

As each treatment was carried out in triplicate, the reproducibility of the microbial community results (both DNA and RNA) was evaluated by means of ANOVA. No significant differences were observed among replicates (*P* < 0.0001), and, as a consequence, reads of replicates were added for the generation of heat maps. Microbial community composition was determined on phylum, class, order, family, and phylotype level (Fig. 2; Additional file 1: Figs. S6–S8). An overall dominance of Bacteroidetes (23.6 ± 5.2%), Proteobacteria (17.2 ± 7.3%), and, to lesser extent, Firmicutes (12.2 ± 3.3%) could be observed in the different reactors, while Euryarchaeota covered on average 4.4 ± 3.1% of the microbial community (Fig. 2a). The Proteobacteria phylum showed a higher relative abundance on RNA (21.1 ± 8.8%) compared with DNA (13.5 ± 2.0%) level, while the opposite was observed for the Bacteroidetes, with relative abundances of 19.9 ± 3.9% on RNA and 27.0 ± 3.7% on DNA level. An increase in Firmicutes in the presence of high salt concentrations could be observed on RNA level, while a decrease in Bacteroidetes could be observed on DNA level in function of time. The Euryarchaeota showed a distinctly lower relative abundance on RNA (2.4 ± 1.0%) compared with DNA level (6.4 ± 3.2%).

While the microbial community profile appeared to be rather uniform at phylum level, this was not the case for the most abundant phylotypes (Fig. 2b). *Methanosaeta* (Phy1), *Rikenella* (Phy2), and two unclassified Bacteroidetes phylotypes (Phy 3 and 4) were each higher on DNA level compared with RNA, and also showed a high level of variation with respect to salt concentration and time. *Methanosaeta* (Phy1) was by far the most abundant methanogen, both on DNA and RNA levels, with the highest relative abundance at low salt concentrations on DNA level, but this was not confirmed on RNA level. The relative abundance of both Phy2 and Phy4 increased on RNA level between day 2 and 7), compared with day 1 and day 14, indicating a higher activity during this period. The relative abundance of Phy15 (unclassified Actinomycetales) showed an increased abundance on RNA level only in the control treatments, indicating its preference for a low salt concentration and/or a competitive advantage over other micro-organisms at these conditions [50].

#### Total and active microbial abundance on community level: organization, variance, and diversity

**Fig. 3:**
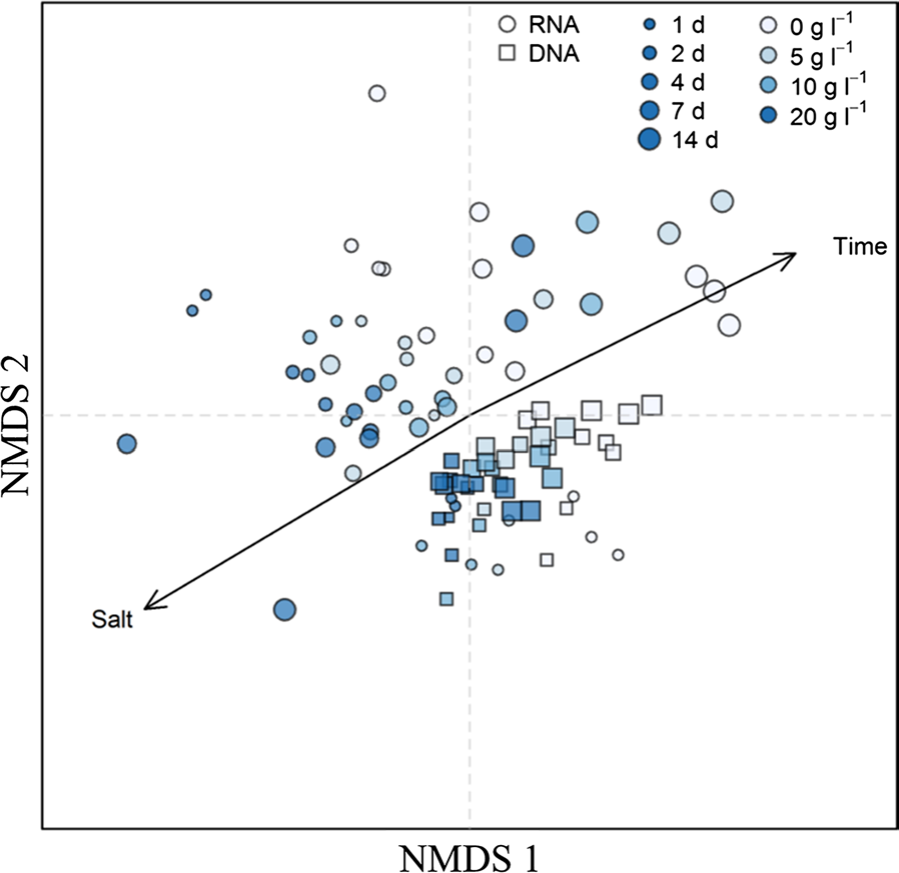
Non-metric multidimensional scaling (NMDS) analysis of the Bray–Curtis dissimilarity index of the microbial community on DNA (□) and RNA (○) level

Beta diversity analysis, including both DNA and RNA samples, revealed a significant correlation with salt concentration (*P* = 0.001) and time (*P* = 0.001), both on the total (DNA) and active (RNA) microbial community (Fig. 3 and Additional file 1: Fig. S9). While the microbial community clustered closely together on DNA level, this appeared not to be the case for the RNA level (Fig. 3). PERMANOVA analysis of the Bray–Curtis dissimilarity index showed a significant difference (*P* = 0.001) between the total and active community. This was confirmed by a significantly different (*P* < 0.0001) overall degree of variation between the DNA and RNA levels, as observed by the relative distance from the centroid, based on the Bray–Curtis dissimilarity index (Additional file 1: Figure S10). The relative distance between the corresponding microbial community abundance and activity profile of each sample was significantly influenced by time (*P* = 0.017), with a significant increase in distance on day 7 (*P* = 0.009) and 14 (*P* = 0.004) of the short-term batch test, while salt concentration did not significantly contribute to this relative distance (*P* = 0.87).

**Fig. 4:**
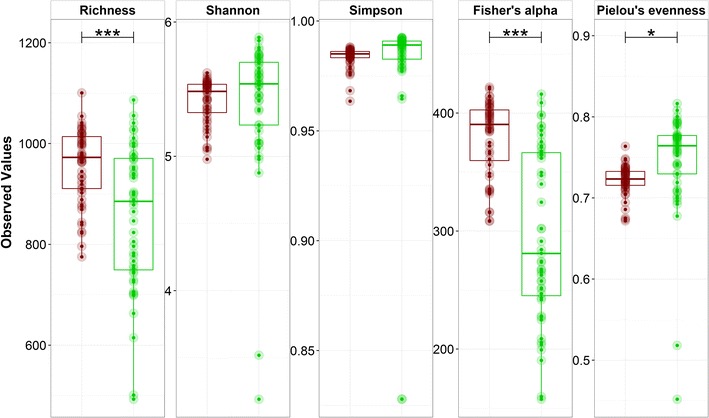
Box plots of the alpha diversity metrics of the microbial community on DNA (*red*) and RNA (*green*) level. Significant differences between the DNA and RNA profile are marked with *(*P* < 0.05), **(*P* < 0.01), or ***(*P* < 0.001), based on the ANOVA analysis

Microbial community diversity analysis revealed an overall significant difference in richness (*P* < 0.0001), Fisher’s alpha diversity (*P* < 0.0001), and Pielou’s evenness (*P* = 0.026) between the DNA and RNA profiles of the different samples (Fig. 4). Richness and Fisher’s alpha diversity were significantly higher on DNA level. In contrast, Pielou’s evenness was significantly higher on RNA level.

Separate analysis on DNA and RNA levels was carried out to estimate the single impact of the salt concentration and time on the total and active microbial community (Additional file 1: Figure S11 and S12). The inoculum sample was excluded from the analysis, as this was only a single sample, to avoid biasing the diversity parameter results. On DNA level, each of the diversity parameters showed a highly significant increase with increasing salt concentration, while this was not the case for the RNA profile for which only Fisher’s alpha diversity and richness showed a minor significant increase (Additional file 1: Figure S11). In contrast to salt concentration, time had a highly significant impact on RNA level, as each of the diversity parameters showed a significant decrease with increasing time, especially for the last two time points (day 7 and 14), with the exception of Simpson diversity (Additional file 1: Figure S12). This was not the case for the DNA profile, with only inconsistent significant differences between the different time points. This clearly reflects the distinct DNA and RNA response, with salt concentration mainly affecting overall relative abundance and time overall activity.

#### Differential impact of salt concentration and time on specific phylotypes

**Fig. 5:**
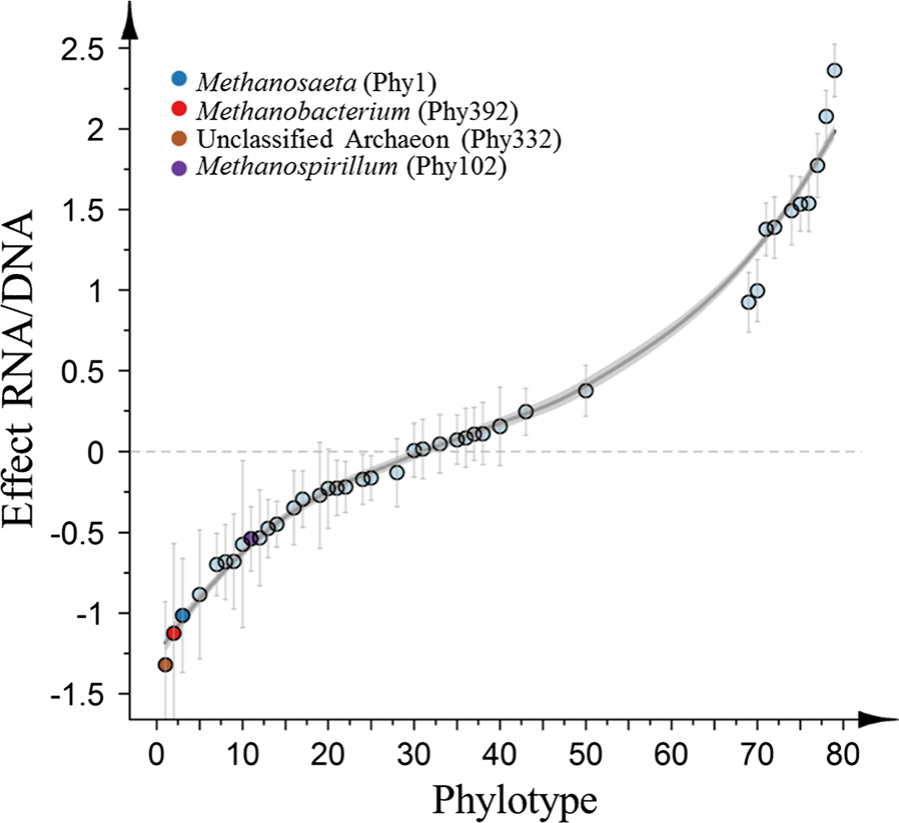
Relative difference in RNA and DNA abundances of the 41 phylotypes that showed a significant difference in RNA and DNA abundances (out of a total of 79 phylotypes). These 79 phylotypes had a relative abundance >0.1% in at least one sample, and were present in all samples. Average values of the parameter estimation of the multivariate abundance model of all samples (excluding the feed sludge samples) are presented, and ordered from the most negative (RNA/DNA ratio <1) to the most positive values (RNA/DNA ratio >1). *Error bars* represent standard errors of the parameter estimation of the model. A smoothing function was fitted to the data for illustration of trends (local regression)

After validation of normality of residuals and homogeneity of variance in the residuals (Additional file 1: Figure S1), multivariate abundance analysis was applied to evaluate the impact of salt concentration and time on the relative abundance of each phylotype between the DNA and RNA levels. To avoid a biased result from low-abundant phylotypes and/or those present in only a limited number of samples, only those phylotypes with a maximum relative abundance >0.1% and present in all DNA and RNA samples were considered for further analysis. This cut-off resulted in 79 phylotypes of which 41 revealed a significant difference (*P* < 0.05) between DNA and RNA relative abundance (Fig. 5). A total of 21 phylotypes showed a significant higher relative abundance on DNA level, while 20 phylotypes showed a higher relative abundance on RNA level. All four archaeal phylotypes with a significant difference in DNA and RNA profile, amongst which *Methanosaeta* (Phy1, *P* = 0.0002), *Methanospirillum* (Phy102, *P* = 0.0002) and *Methanobacterium* (Phy392, *P* = 0.0004) showed a lower relative abundance on RNA level, which was also the case for *Rikenella* (Phy2, *P* = 0.0002) and other phylotypes belonging to the Bacteroidetes phylum. In contrast, two unclassified Actinomycetales (Phy 15 and 27, *P* = 0.0002), two unclassified Armatimonadetes (Phy49 and 63, *P* = 0.0002), and an unclassified Synergistaceae (Phy28, *P* = 0.0002) showed a significant higher relative abundance on RNA level.

**Fig. 6:**
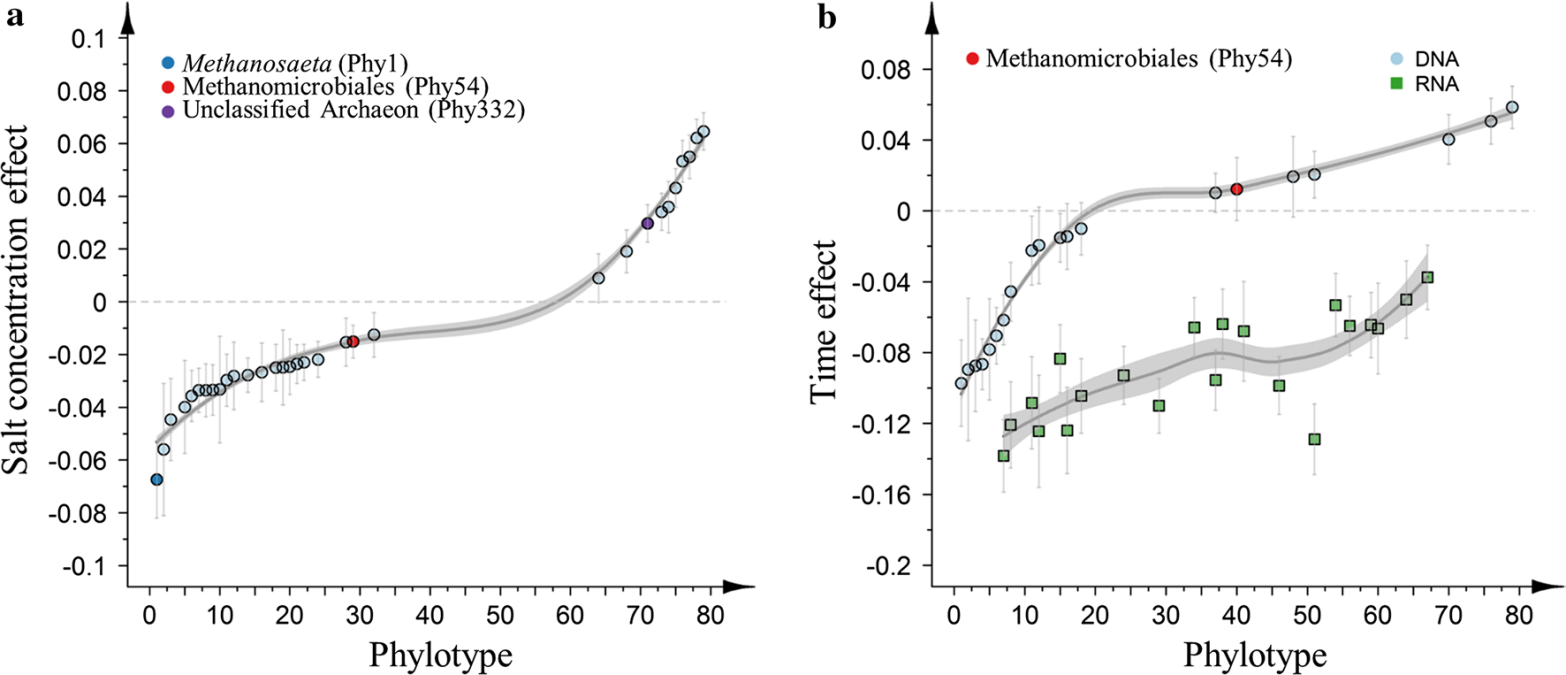
Relative effect of **a** salt concentration and **b** time on the 32 and 20 phylotypes (●) that showed a significant different abundance in function of salt concentration and time, respectively (out of a total of 79 phylotypes). These phylotypes had minimum relative abundance >0.1% in at least one sample, and were present in all samples. Average values of the parameter estimation of the multivariate abundance model of all samples (excluding the feed sludge samples) are presented, and ordered from the most negative (negative effect) to the most positive (positive effect) values. The effect strength of time was additionally dependent on the RNA/DNA ratio for 21 phylotypes (■), while this could only be observed for two phylotypes for the salt effect (not included in the figure). *Error bars* represent standard errors of the parameter estimation of the model. A smoothing function was fitted to the data for illustration of trends (local regression)

Both salt concentration and time also affected specific phylotypes, with 32 and 20 phylotypes significantly influenced by salt concentration and time, respectively (Fig. 6; Additional file 1: Table S4). Salt concentration had the strongest negative effect on *Methanosaeta* (Phy1, *P* = 0.024), and also an unclassified Methanomicrobiales phylotype (Phy54, *P* = 0.031) was negatively affected by salt, while an unclassified Archaeon (Phy685, *P* = 0.012) was positively affected by salt. Time had a significant positive effect on the unclassified Methanomicrobiales phylotype (Phy54, *P* = 0.008).

**Fig. 7:**
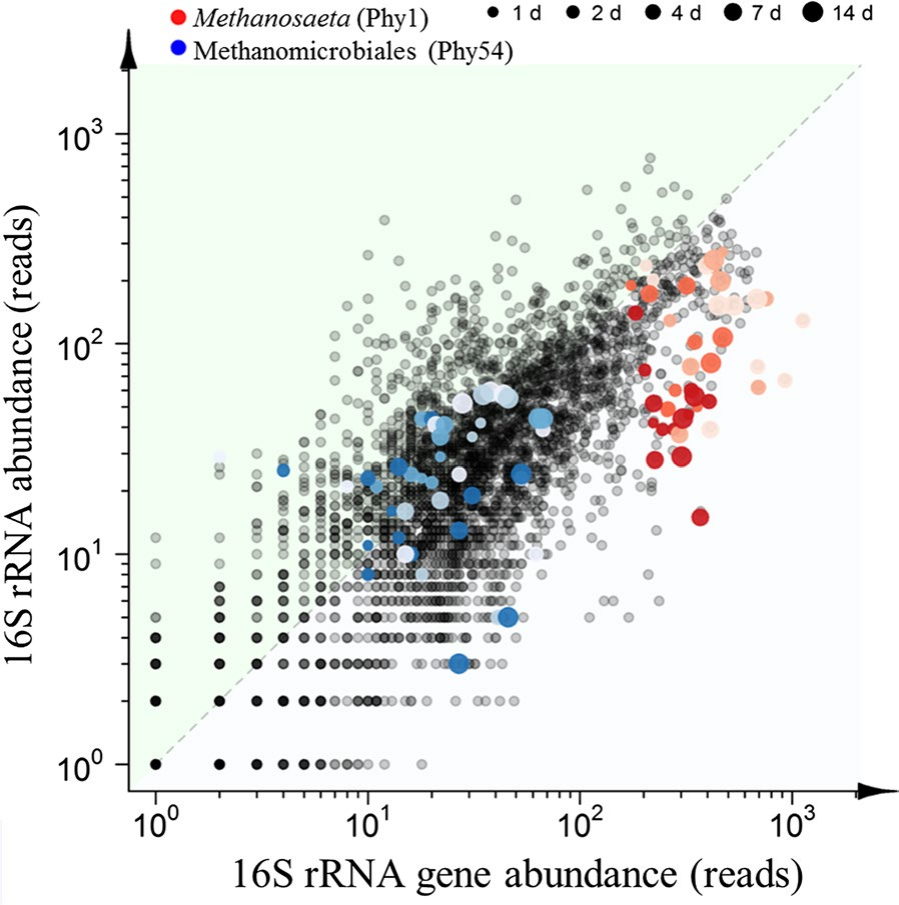
Comparison of RNA and DNA relative abundances of the selected 79 phylotypes in each of the samples (excluding the feed sludge sample). These 79 phylotypes had a relative abundance >0.1% in at least one sample and were present in all samples. The two most abundant methanogens [*Methanosaeta*, Phy1 (*red*) and an unclassified Methanomicrobiales phylotype, Phy54 (*blue*)] were highlighted, and the intensity of the colour relates to the salt concentration. The *dashed line* represents a DNA:RNA relative abundance ratio of 1

The effect strength of time was additionally dependent on the RNA/DNA ratio for 21 phylotypes, while this could only be observed for 2 phylotypes for the salt effect (Fig. 6; Additional file 1: Table S4). This indicates a stronger effect of time on the RNA/DNA ratio of specific phylotypes compared with salt concentration. The two main archaeal phylotypes (*i.e. Methanosaeta*, Phy1 and unclassified Methanomicrobiales, Phy54) clearly showed a different response on RNA and DNA levels and with respect to salt concentration and time (Figs. 5 and 6). This was confirmed by a visualization of the 79 selected phylotypes (Fig. 7). A significantly lower RNA/DNA ratio could be observed for *Methanosaeta* (*P* = 0.0002), with the strongest decrease at high salt concentrations. The unclassified Methanomicrobiales did not show a change in RNA/DNA ratio over time or related to salt, or difference in relative abundance on RNA and DNA levels, but, nonetheless, a significant negative (*P* = 0.031) effect was observed in relation to salt, while there was a significant positive effect of time (*P* = 0.008). Overall, the methanogenic phylotypes showed the strongest differential response between DNA and RNA, both to salt concentration and time.

## Discussion

The introduction of salt perturbation in a short-term AD batch test resulted in a distinct negative effect on methane production for the highest salt concentrations. A clear differentiation in terms of overall composition, variation, and diversity was observed between the total (DNA) and active microbial community (RNA), related to both salt concentration and time. The multivariate regression approach allowed to determine not only the phylotypes that experienced a significant effect of salt concentration and time, but also the size of the effect. This was reflected in the differential response of specific phylotypes, amongst which the dominant archaeon *Methanosaeta* experienced the strongest effect, while the effect was less pronounced for most bacterial phylotypes.

### Salt concentration and time differentially impact the total and active microbial community

The comparison of the microbial community via DNA- and RNA-based measurements revealed an overall higher diversity on DNA level, and a higher degree of variation on RNA level. A high species richness and diversity can relate with an increased resilience, as a larger pool of available micro-organisms enhances the potential to maintain functionality, and respond to disturbances [14, 16, 51]. Such a high community compositional diversity does not necessarily reflect a similar profile in terms of activity. In principle, few species are required to take care of the entire AD process, while the others are dormant, and will not become active before a change in environmental conditions occurs [52, 53]. This was also the case for the results of this study, as a lower richness is observed on RNA level, while the higher evenness relates with a higher degree of functionality [12–14, 54], although there is no direct dependency of functionality on microbial richness and evenness. As there is no direct proof for the relation between microbial richness or evenness and process stability, these results should be interpreted with care.

Salt perturbation often promotes a change in microbial community composition and/or organization [55, 56], which reflects the susceptibility of certain micro-organisms and the resistance of others to salt perturbation. The remarkable difference in diversity parameters between DNA and RNA in relation to both salt concentration and time, as observed in this study, reflects the different mechanisms that drive the total and active microbial community profiles. The significant increase in diversity on DNA level in relation to increasing salt concentrations was not observed on RNA level. This can be explained by the combined effect of the apparent microbial community shift and the difference in half-life between DNA and RNA molecules. The increased salt concentration resulted in a shift in microbial community composition, as observed by beta diversity analysis. This led to other species becoming more active in detriment of others that became less active or even dormant, thus explaining the limited degree of variation in diversity on RNA level. However, due to the stability of the DNA molecule compared with RNA and the fact that a decrease in activity does not lead to a decrease in abundance on short-term, diversity on DNA level increased. The decrease in diversity on RNA level in function of time related to the decrease in methane production at the end of the test, because only a single feed pulse was introduced at the beginning of the test.

The higher degree of overall variation on RNA level could not be attributed to the salt concentration. This unexplainable variation on RNA level indicates that the selection of active species depends both on deterministic and stochastic processes. Deterministic factors, such as pH and temperature, determine which species can be present (DNA) in the anaerobic digester [57, 58], while the actual active community (RNA) seems to be influenced mainly by stochastic processes, related to the apparent random variation on RNA level [59].

### Specific phylotypes determine the overall microbial community response to salt perturbation

The active microbial community in AD seems to aim for a high degree of evenness, regardless the lower level of evenness in the total community, which undoubtedly relates with the requisite for a well-balanced succession of the different phases in the AD process. This high degree of evenness on RNA level is, however, insufficient to assure stable process performance in AD, because methane production declined nonetheless at high salt concentrations. This implies the importance of specific active (groups of) micro-organisms to ensure process stability.

Microbial community analysis on different phylogenetic levels revealed an overall uniform pattern on the higher phylogenetic levels in relation to salt concentration and time, while a much stronger degree of variation could be observed between different phylotypes. The overall dominance of the Bacteroidetes, Proteobacteria, and Firmicutes bacterial phyla is to be expected, given the versatile consortia of bacteria in these phyla involved in different phases of the AD process [18, 21, 60]. The overall higher activity of the Proteobacteria phylum can be considered an indication of their higher involvement than conventionally estimated in the AD process. However, due to the metabolic diversity of the different members of this phylum, the overall importance of this phylum in AD cannot be confirmed. More important for the AD process is the lower relative abundance of Euryarchaeota on RNA level, which can be attributed almost entirely to the decrease in *Methanosaeta* (Phy1), as this phylotype covered on average 80% of archaeal reads, both on DNA and RNA levels. Next to *Methanosaeta*, several bacterial dominant phyla, such as *Rikenella* (Phy2), also showed a clear decrease on RNA compared with DNA level, which also explains the higher evenness on RNA level.

### Acetoclastic methanosaeta as crucial active methanogen in relation to salt perturbation

The overall importance of *Methanosaeta* for methane production in the AD process has been confirmed by numerous studies, as well as its susceptibility to changes in environmental conditions outside the optimal range for methane production [18, 34, 61, 62]. However, this is the first study in which the relative abundance of *Methanosaeta* in AD is compared between the DNA and RNA levels. The RNA profile of *Methanosaeta* (Phy1) suggests a lower activity than estimated by its DNA profile, but this is similar for several other methanogenic phylotypes. This is in contrast with other studies in which a higher (potential) activity in relation to the DNA level was observed [24–26]. In this study, the lower relative abundance of *Methanosaeta* on RNA level related to the increase in salt concentration, which was not the case for the other methanogens, with the exception of an unclassified Methanomicrobiales phylotype (Phy54), indicating that salt perturbation strongly affects *Methanosaeta* in AD. The persistence of a high abundance of *Methanosaeta* at suboptimal conditions [63] does not necessarily reflect an equally high activity, as observed in our study. Due to its high susceptibility to environmental changes, *Methanosaeta* is often replaced by *Methanosarcina* as primordial acetoclastic methanogen [64], but in this study, no phylotypes related to *Methanosarcina* could be detected. This may explain the inhibition of methane production at 10 and 20 g Na^+^ L^−1^, as the hydrogenotrophic methanogens were unable to take over the methane production process. None of the observed known syntrophic acetate oxidizing bacteria, which are necessary to redirect acetate conversion from acetoclastic to hydrogenotrophic methanogenesis, showed an increase in activity or abundance in relation to salt perturbation [65, 66].

The crucial role of *Methanosaeta* in AD is apparent, which is amplified by the fact that in the absence of *Methanosarcina* and/or a sufficient response of potential syntrophic acetate oxidizing bacteria, the system collapses if *Methanosaeta* is not able to maintain its activity. Hence, despite its low relative abundance on RNA level in this study, *Methanosaeta* can be considered the most important methanogen in AD to obtain stable methane production.

## Conclusions

This research demonstrates a different response between the total (DNA) and active (RNA) microbial community in AD with respect to salt perturbation. Salt concentration and time more strongly affected the microbial community, and especially the archaeal phylotypes, on RNA level both in terms of overall diversity and specific phylotypes. The most abundant archaeal phylotype, *Methanosaeta*, showed a lower relative abundance on RNA level compared with DNA level, caused by salt perturbation. Although microbial community activity analysis based on the rRNA profile should be interpreted with care, given its limitations [67], a clear differentiation between the RNA and DNA profile of the microbial community was observed in this research. This confirms the overall importance of RNA-based community screening to analyse the response to disturbances and to identify those micro-organisms potentially contributing to process performance and stability in AD. However, the limitations of 16S rRNA gene sequences in terms of differences in 16S rRNA gene copy numbers between species have to be considered, and only major changes could be identified based on changes in the 16S rRNA (gene). Nonetheless, these results proved to be valuable with respect to the relation between community changes and process disturbance, and could be used to develop suitable indicators to engage in more accurate control of the AD process.

## Additional files

**Additional file 1: Table S1.** Characteristics of the substrate used for the mother reactors and the short-term stress test. Each analysis was carried out in triplicate, except for the cation measurements. **Table S2** Characteristics of the inoculum used for the mother reactors. Each analysis was carried out in triplicate. **Table S3** Characteristics of the inoculum used for the short-term stress test. This inoculum originated from the mixture of the three mother reactors. Each analysis was carried out in triplicate. **Table S4** Overview of the *P*-values of the multivariate abundance model of the 79 phylotypes that had a relative abundance >0.1% in at least one sample, and were present in all samples. Adjusted *P*-values were determined for salt concentration, time, and the DNA/RNA ratio, as well as the interaction effect between salt concentration and DNA/RNA ratio and time and DNA/RNA ratio. Highlighted *P*-values are considered significant at α = 0.05. **Table S5** Overview of the model coefficients of the multivariate abundance model of the 79 phylotypes that had a relative abundance >0.1% in at least one sample, and were present in all samples. Coefficients were determined for salt concentration, time, and the DNA/RNA ratio, as well as the interaction effect between salt concentration and DNA/RNA ratio and time and DNA/RNA ratio. A negative value indicates a negative effect of salt or time or a DNA/RNA ratio > 1. **Fig. S1**. Evaluation of model assumptions required to allow correct inference of the multivariate regression model with (a) residuals vs. fitted, (b) scale location, and (c) normal Q–Q results. Each label colour is associated with a single phylotype. These results show that the normality of residuals and homogeneity of variance requirements are fulfilled. **Fig. S2** Overview of the main operational parameters of the triplicate mother reactors, including (a) methane production, (b) pH of the reactors (■) and the substrate (●), (c) total VFA, and (d) partial alkalinity (■), total alkalinity (●), and the Ripley index (▲). **Fig. S3** Cumulative methane yield of the control treatment (□), and treatments with 5 g Na^+^ L^-1^ (○), 10 g Na^+^ L^-1^ (■), and 20 g Na^+^ L^-1^ (●). **Fig. S4** Total VFA, and pH and Na+ concentration in the reactors in the stress test after 14 days of operation. Average values of the triplicate reactors are presented, and error bars show standard deviations. **Fig. S5** Rarefaction curves indicating the number of resolved phylotypes against sampling depth of each of the samples (both DNA and RNA) during the 14 days short-term stress test. **Fig. S6** Heat map representing the microbial community of all samples on class level at a relative abundance >0.1% averaged over all samples. The colour scale ranges from 0 to 60%. The different samples were labelled according to the time point (T0–T5) and salt concentration (0, 5, 10 and 20 g Na^+^ L^-1^). The inoculum sample (Inoc) at the start of the experiment as well as the feed sludge (Sludge) were also included in the heat map. **Fig. S7** Heat map representing the microbial community of all samples on order level at a relative abundance >0.1% averaged over all samples. The colour scale ranges from 0 to 70%. The different samples were labelled according to the time point (T0–T5) and salt concentration (0, 5, 10 and 20 g Na^+^ L^-1^). The inoculum sample (Inoc) at the start of the experiment as well as the feed sludge (Sludge) were also included in the heat map. **Fig. S8** Heat map representing the microbial community of all samples on family level at a relative abundance >0.1% averaged over all samples. The colour scale ranges from 0 to 60%. The different samples were labelled according to the time point (T0–T5) and salt concentration (0, 5, 10 and 20 g Na^+^ L^-1^). The inoculum sample (Inoc) at the start of the experiment as well as the feed sludge (Sludge) were also included in the heat map. **Figure S9** Non-metric multidimensional scaling analysis of the Bray–Curtis dissimilarity index of the different samples on (a) DNA and (b) RNA levels. **Figure S10** Overall microbial community variation between the different samples on DNA (red) and RNA (green) level, determined by the distance to the centroid. The distance to the centroid is significantly higher for the RNA samples than the DNA samples (*P* < 0.0001). **Figure S11** Box plots of the alpha diversity metrics on (a) DNA and (b) RNA levels for the different salt additions, ranging from 0 (Control) to 20 g Na^+^ L^-1^. Samples were divided in subgroups per salt concentration for comparison of the alpha diversity metrics. A significant difference between any two salt concentrations is marked with * (*P* < 0.05), **(*P* < 0.01), or ***(*P* < 0.001), based on the ANOVA analysis. **Figure S11** Box plots of the alpha diversity metrics on (a) DNA and (b) RNA levels for the different salt additions, ranging from 0 (Control) to 20 g Na^+^ L^-1^. Samples were divided in subgroups per salt concentration for comparison of the alpha diversity metrics. A significant difference between any two salt concentrations is marked with *(*P* < 0.05), **(*P* < 0.01), or ***(*P* < 0.001), based on the ANOVA analysis.

**Additional file 2.**

## Abbreviations

ANOVA: analysis of variance
AD: anaerobic digestion
COD: chemical oxygen demand
IC: ion chromatography
PCR: polymerase chain reaction
PERMANOVA: permutational multivariate analysis of variance
STP: standard temperature (273.15 K) and pressure (101,325 Pa)
TAN: total ammonia nitrogen
TS: total solids
VFA: volatile fatty acids
VS: volatile solids
