# Supplementary material for: Presence does not imply activity: DNA and RNA patterns differ in response to salt perturbation in anaerobic digestion
Source: Biotechnol Biofuels. 2016 Nov 9;9:244. doi: 10.1186/s13068-016-0652-5 (PMC5103597; doi:10.1186/s13068-016-0652-5)
Supplement: Supplementary file 1 — Additional file 1: Table S1. Characteristics of the substrate used for the mother reactors and the short-term stress test. Each analysis was carried out in triplicate, except for the cation measurements. Table S2 Characteristics of the inoculum used for the mother reactors. Each analysis was carried out in triplicate. Table S3 Characteristics of the inoculum used for the short-term stress test. This inoculum originated from the mixture of the three mother reactors. Each analysis was carried out in triplicate. Table S4 Overview of the P-values of the multivariate abundance model of the 79 phylotypes that had a relative abundance >0.1% in at least one sample, and were present in all samples. Adjusted P-values were determined for salt concentration, time, and the DNA/RNA ratio, as well as the interaction effect between salt concentration and DNA/RNA ratio and time and DNA/RNA ratio. Highlighted P-values are considered significant at α = 0.05. Table S5 Overview of the model coefficients of the multivariate abundance model of the 79 phylotypes that had a relative abundance >0.1% in at least one sample, and were present in all samples. Coefficients were determined for salt concentration, time, and the DNA/RNA ratio, as well as the interaction effect between salt concentration and DNA/RNA ratio and time and DNA/RNA ratio. A negative value indicates a negative effect of salt or time or a DNA/RNA ratio > 1. Fig. S1. Evaluation of model assumptions required to allow correct inference of the multivariate regression model with (a) residuals vs. fitted, (b) scale location, and (c) normal Q–Q results. Each label colour is associated with a single phylotype. These results show that the normality of residuals and homogeneity of variance requirements are fulfilled. Fig. S2 Overview of the main operational parameters of the triplicate mother reactors, including (a) methane production, (b) pH of the reactors (■) and the substrate (●), (c) total VFA, and (d) partial alkalinity (■), tot [file 13068_2016_652_MOESM1_ESM.docx]

**Supporting Information**

Title: Presence does not imply activity: DNA and RNA patterns differ in response to salt perturbation in anaerobic digestion.

**Jo De Vrieze^1,2*^, Leticia Regueiro^2*^, Ruben Props^1^, Ramiro Vilchez-Vargas^1^, Ruy Jáuregui^3^, Dietmar H. Pieper^3^, Juan M. Lema^2^, Marta Carballa^2🖂^**

^1^Center for Microbial Ecology and Technology (CMET), Ghent University, Coupure Links 653, B-9000 Gent, Belgium

^2^Department of Chemical Engineering, School of Engineering, University of Santiago de Compostela, Rúa Lope Gomez de Marzoa s/n, E-15782 Santiago de Compostela, Spain

^3^Microbial Interactions and Processes Research Group, Helmholtz Centre for Infection Research (HZI), Braunschweig, Germany

^a^Both authors contributed equally to this work.

^b^Current address: AgResearch, Tennent Drive, Palmerston North 4442, New Zealand

**^🖂^** Correspondence to: Jo De Vrieze, Ghent University; Faculty of Bioscience Engineering; Center for Microbial Ecology and Technology (CMET); Coupure Links 653; B-9000 Gent, Belgium; phone: +32 (0)9 264 59 76; fax: +32 (0)9 264 62 48; E-mail: Jo.DeVrieze@UGent.be; Webpage: www.cmet.ugent.be.

Contents

[Contents 2](#_Toc454272130)

[S1. Substrate characteristics 3](#_Toc454272131)

[S2. Characteristics of the inoculum of the mother reactor 4](#_Toc454272132)

[S3. Characteristics of the inoculum of the short-term stress test 5](#_Toc454272133)

[S4. Multivariate abundance analysis: evaluating model assumptions 6](#_Toc454272134)

[S5. Mother reactor operation 7](#_Toc454272135)

[S6. Short-term stress test operation 8](#_Toc454272136)

[S7. Rarefaction curves 10](#_Toc454272137)

[S8. Heatmaps 11](#_Toc454272138)

[S9. Beta diversity analysis 14](#_Toc454272139)

[S10. Community variation between DNA and RNA 15](#_Toc454272140)

[S11. Alpha diversity parameters 16](#_Toc454272141)

# S1. Substrate characteristics

**Table S1** Characteristics of the substrate used for the mother reactors and the short-term stress test. Each analysis was carried out in triplicate, except for the cation measurements.

| Parameter |  |
| --- | --- |
| pH | 5.65 ± 0.19 |
| Total solids (g L^-1^) | 30.0 ± 0.1 |
| Volatile solids (g L^-1^) | 21.5 ± 0.2 |
| Total COD (g COD L^-1^) | 43.3 ± 2.9 |
| Total VFA (mg COD L^-1^) | 2970 ± 1153 |
| Acetate (mg COD L^-1^) | 1169 ± 351 |
| Propionate (mg COD L^-1^) | 970 ± 318 |
| Total ammonia (mg N L^-1^) | 81 ± 1 |
| Na^+^ (mg L^-1^) | 72 |
| K^+^ (mg L^-1^) | 111 |
| Ca^+^ (mg L^-1^) | 66 |
| Mg^+^ (mg L^-1^) | 49 |
| TS:VS | 1.40 ± 0.02 |
| COD:VS | 2.02 ± 0.14 |

# S2. Characteristics of the inoculum of the mother reactor

**Table S2** Characteristics of the inoculum used for the mother reactors. Each analysis was carried out in triplicate.

| Parameter |  |
| --- | --- |
| Total solids (g L^-1^) | 273.9 ± 2.2 |
| Volatile solids (g L^-1^) | 113.5 ± 2.5 |
| pH | 7.22 ± 0.09 |
| Total VFA (mg COD L^-1^) | 1561 ± 202 |
| Acetate (mg COD L^-1^) | 1050 ± 170 |
| Propionate (mg COD L^-1^) | 445 ± 38 |
| Total ammonia (mg N L^-1^) | 1330 ± 25 |
| Free ammonia (mg N L^-1^) | 23 ± 5 |
| Total alkalinity (g CaCO_3_ L^-1^) | 1382 ± 319 |
| Partial alkalinity (g CaCO_3_ L^-1^) | 1013 ± 160 |

# S3. Characteristics of the inoculum of the short-term stress test

**Table S3** Characteristics of the inoculum used for the short-term stress test. This inoculum originated from the mixture of the three mother reactors. Each analysis was carried out in triplicate.

| Parameter |  |
| --- | --- |
| Total solids (g L^-1^) | 59.1 ± 2.0 |
| Volatile solids (g L^-1^) | 19.9 ± 0.8 |
| pH | 7.06 ± 0.02 |
| Total VFA (mg COD L^-1^) | 0 ± 0 |
| Acetate (mg COD L^-1^) | 0 ± 0 |
| Propionate (mg COD L^-1^) | 0 ± 0 |
| Total ammonia (mg N L^-1^) | 671 ± 83 |
| Free ammonia (mg N L^-1^) | 8 ± 1 |
| Total alkalinity (mg CaCO_3_ L^-1^) | 2425 ± 53 |
| Partial alkalinity (mg CaCO_3_ L^-1^) | 1873 ± 53 |
| Na^+^ (mg L^-1^) | 240 ± 12 |
| K^+^ (mg L^-1^) | 98 ± 10 |

# S4. Multivariate abundance analysis: evaluating model assumptions


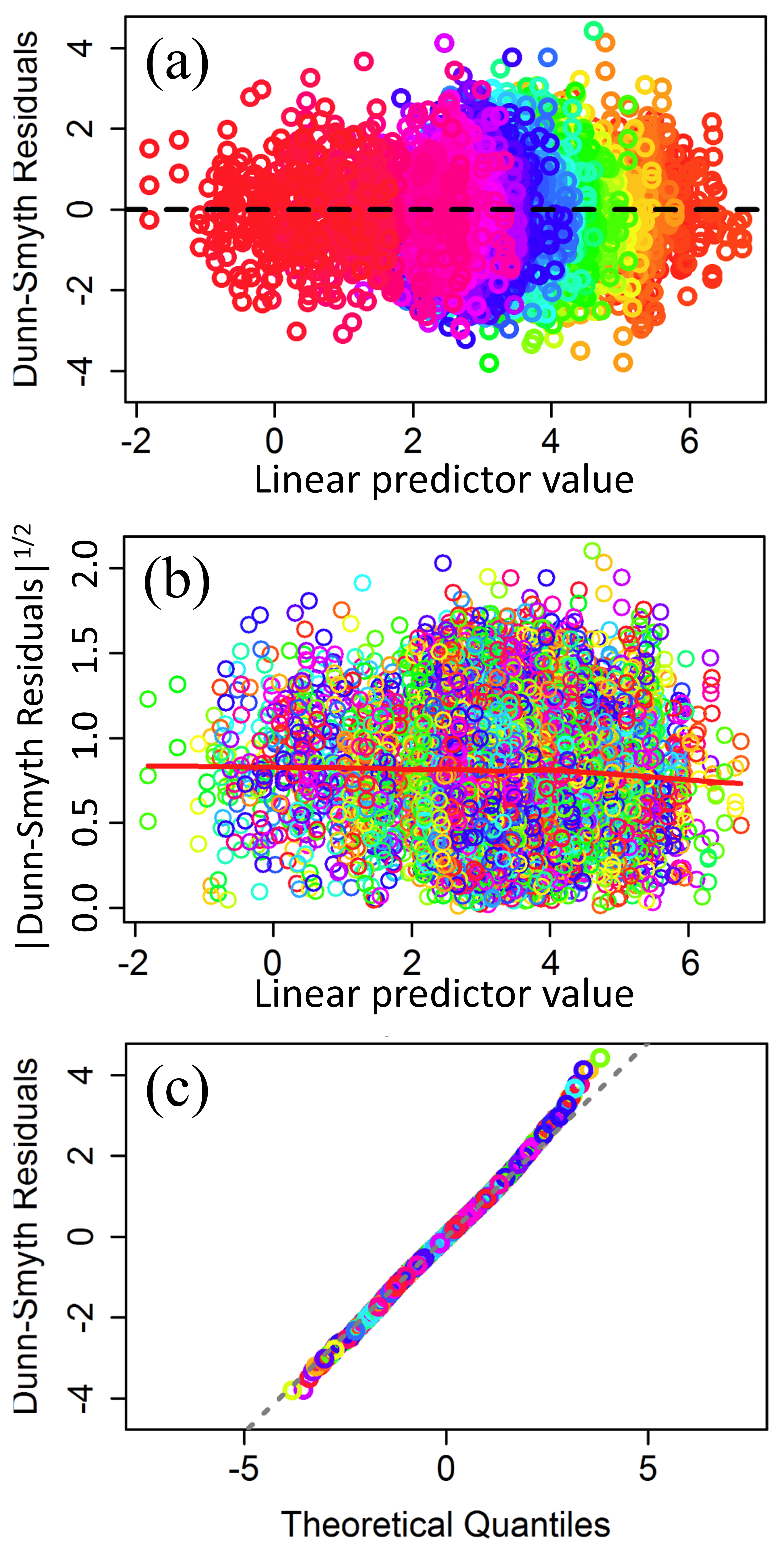


Figure S1 Evaluation of model assumptions required to allow correct inference of the multivariate regression model with (a) residuals vs. fitted, (b) scale location and (c) normal Q-Q results. Each label colour is associated with a single phylotype. These results show that the normality of residuals and homogeneity of variance requirements are fulfilled.

# S5. Mother reactor operation


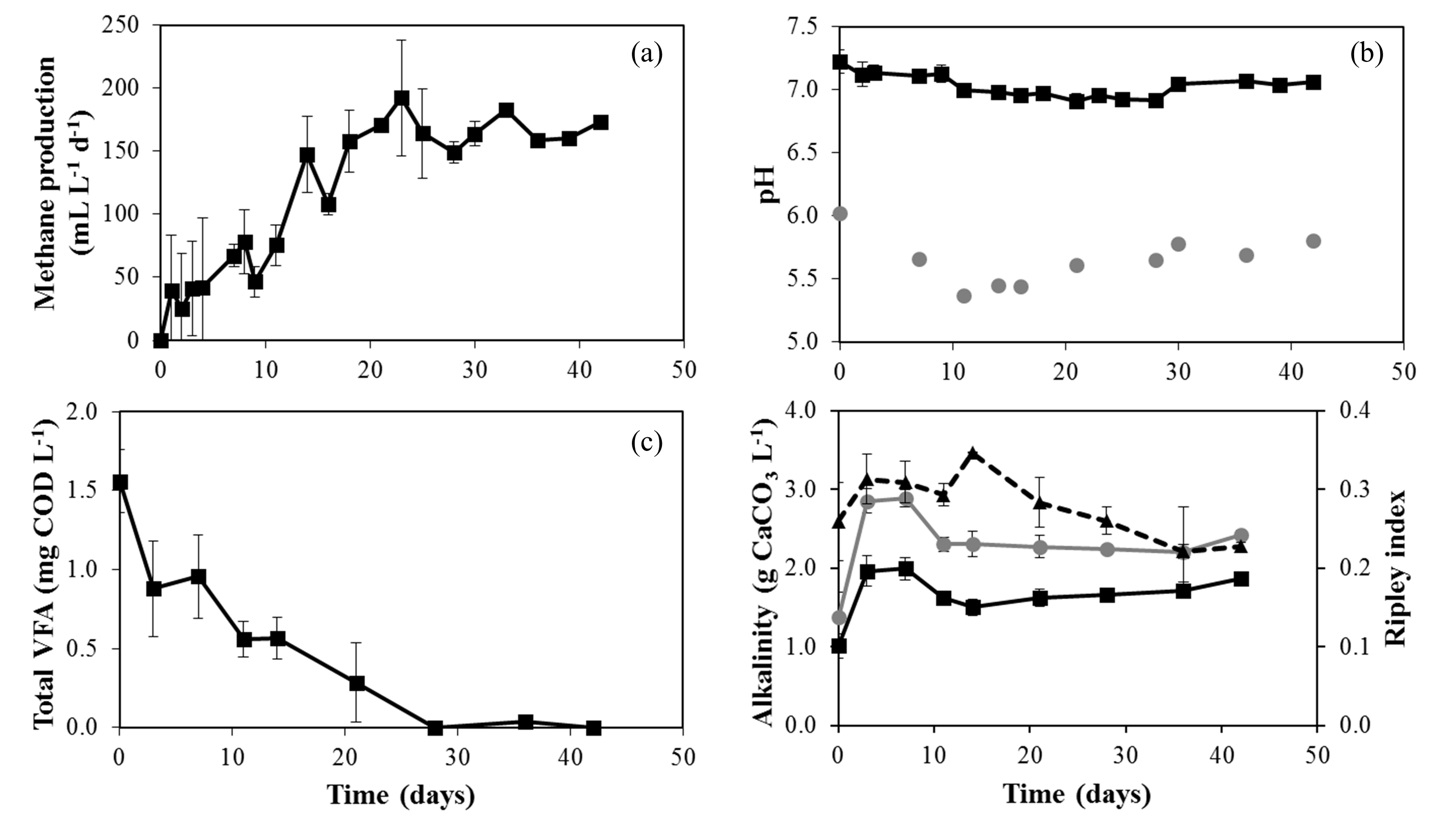


**Figure S2** Overview of the main operational parameters of the triplicate mother reactors, including (a) methane production, (b) pH of the reactors (■) and the substrate (●), (c) total VFA, and (d) partial alkalinity (■), total alkalinity (●), and the Ripley index (▲).

# S6. Short-term stress test operation


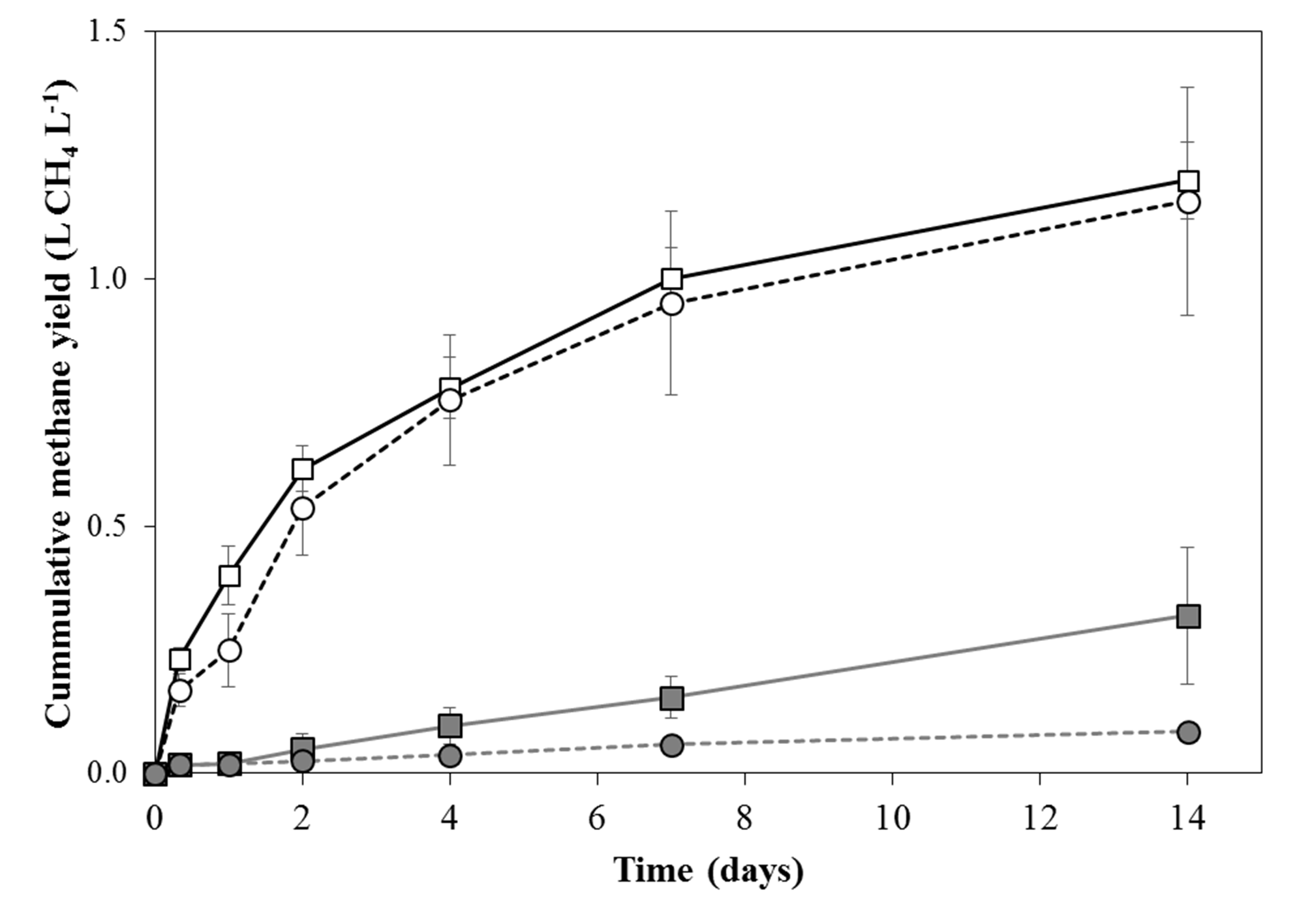


**Figure S3** Cumulative methane yield of the control treatment (□), and treatments with 5 g Na^+^ L^-1^ (○), 10 g Na^+^ L^-1^ (■), and 20 g Na^+^ L^-1^ (●).


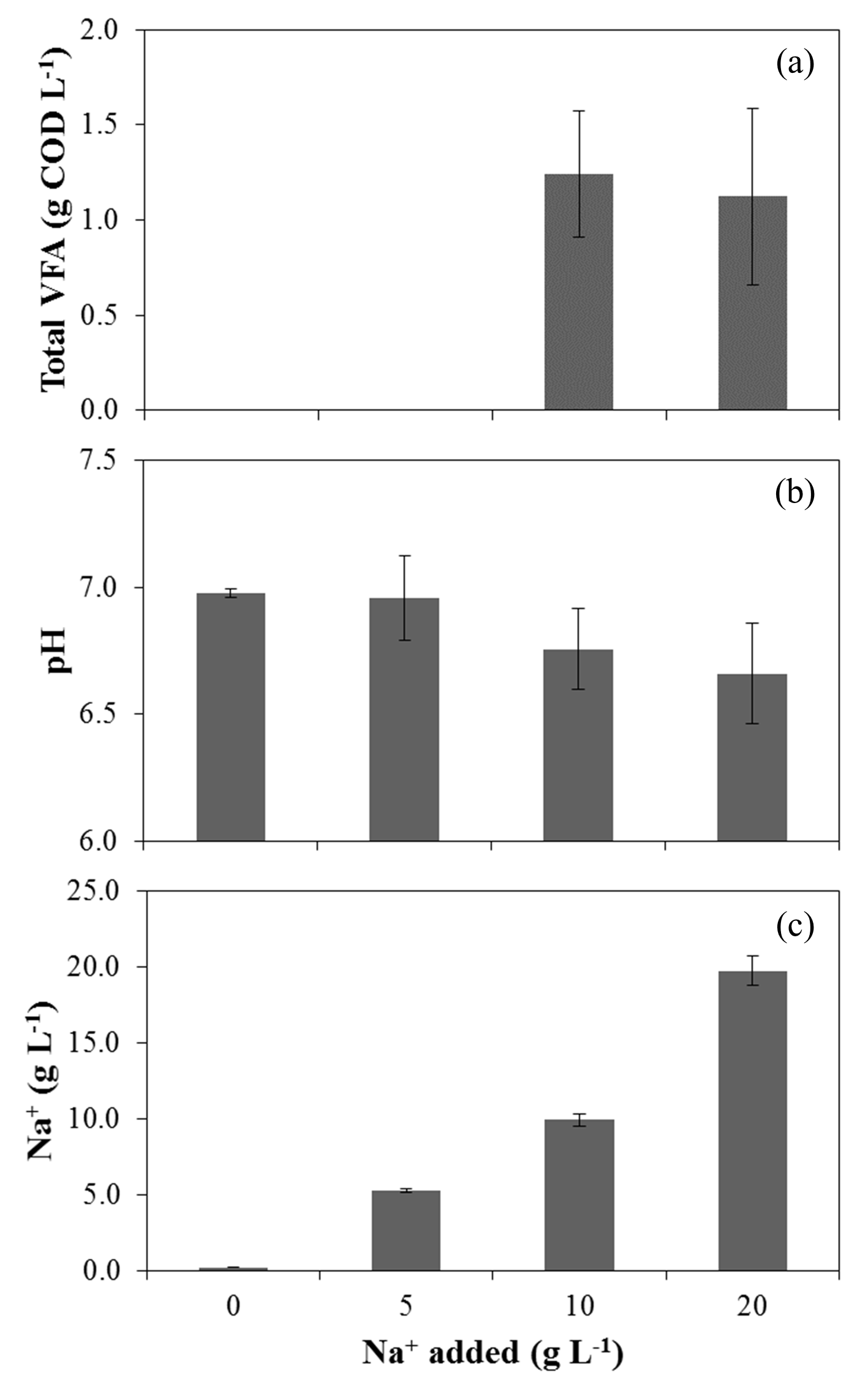


**Figure S4** Total VFA, pH and Na+ concentration in the reactors in the stress test after 14 days of operation. Average values of the triplicate reactors are presented, and error bars show standard deviations.

# S7. Rarefaction curves


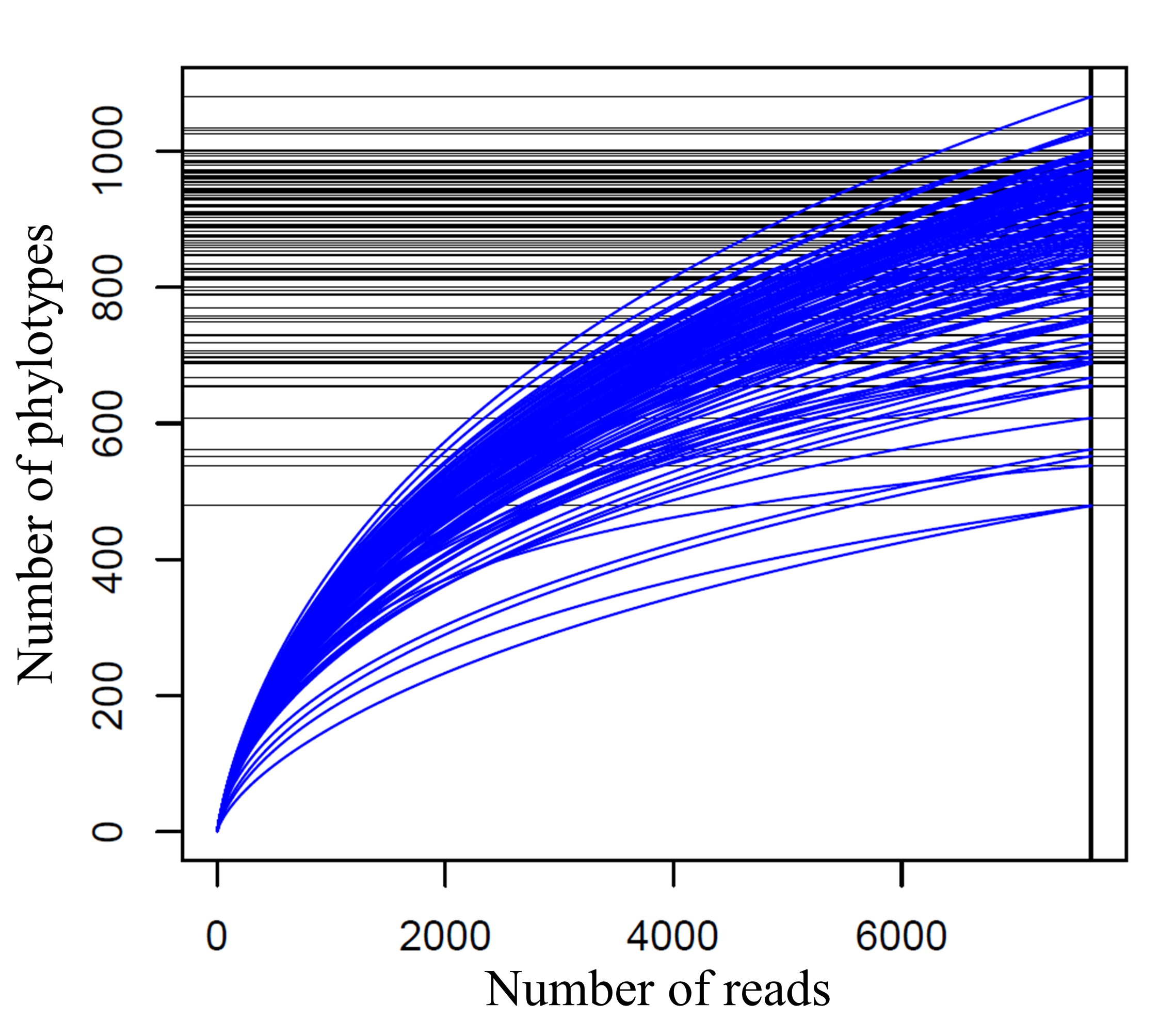


**Figure S5** Rarefaction curves indicating the number of resolved phylotypes against sampling depth of each of the samples (both DNA and RNA) during the 14-days short-term stress test.

# S8. Heatmaps


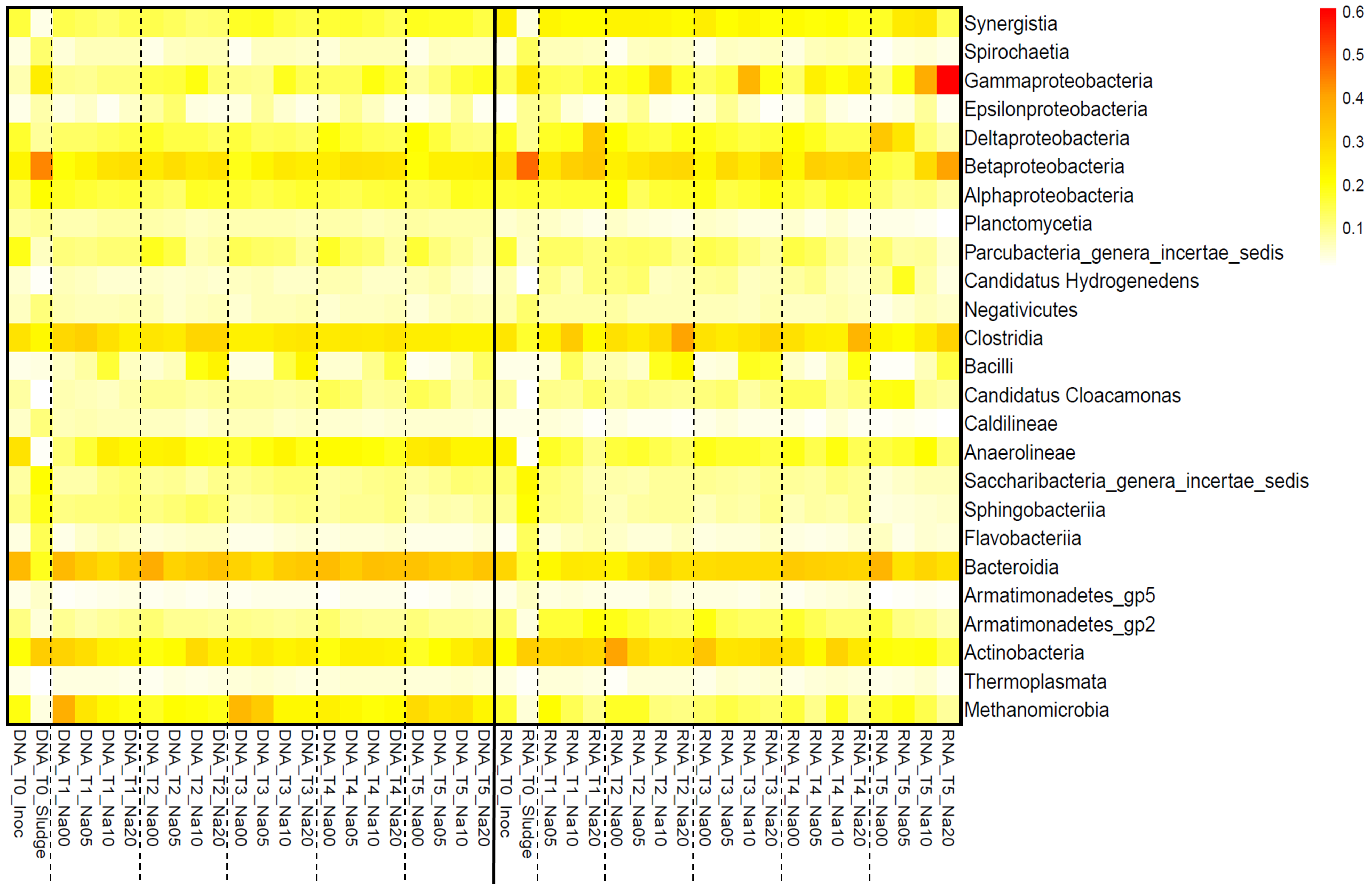


**Figure S6** Heatmap representing the microbial community of all samples on class level at a relative abundance > 0.1% averaged over all samples. The colour scale ranges from 0 to 60%. The different samples were labelled according to the time point (T0-T5) and salt concentration (0, 5, 10 and 20 g Na^+^ L^-1^). The inoculum sample (Inoc) at the start of the experiment, as well as the feed sludge (Sludge) were also included in the heatmap.

**
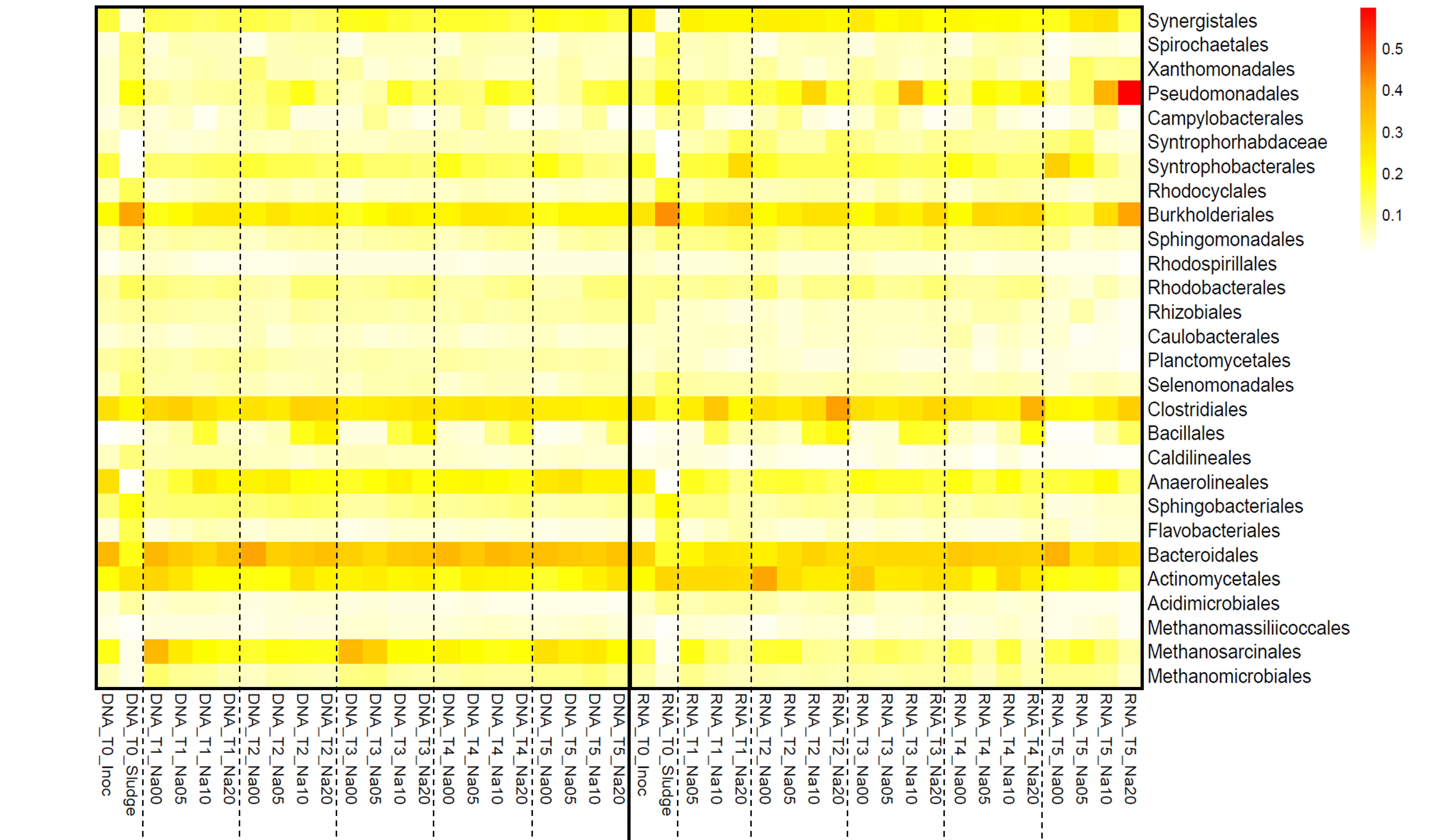
**

**Figure S7** Heatmap representing the microbial community of all samples on order level at a relative abundance > 0.1% averaged over all samples. The colour scale ranges from 0 to 70%. The different samples were labelled according to the time point (T0-T5) and salt concentration (0, 5, 10 and 20 g Na^+^ L^-1^). The inoculum sample (Inoc) at the start of the experiment, as well as the feed sludge (Sludge) were also included in the heatmap.

**
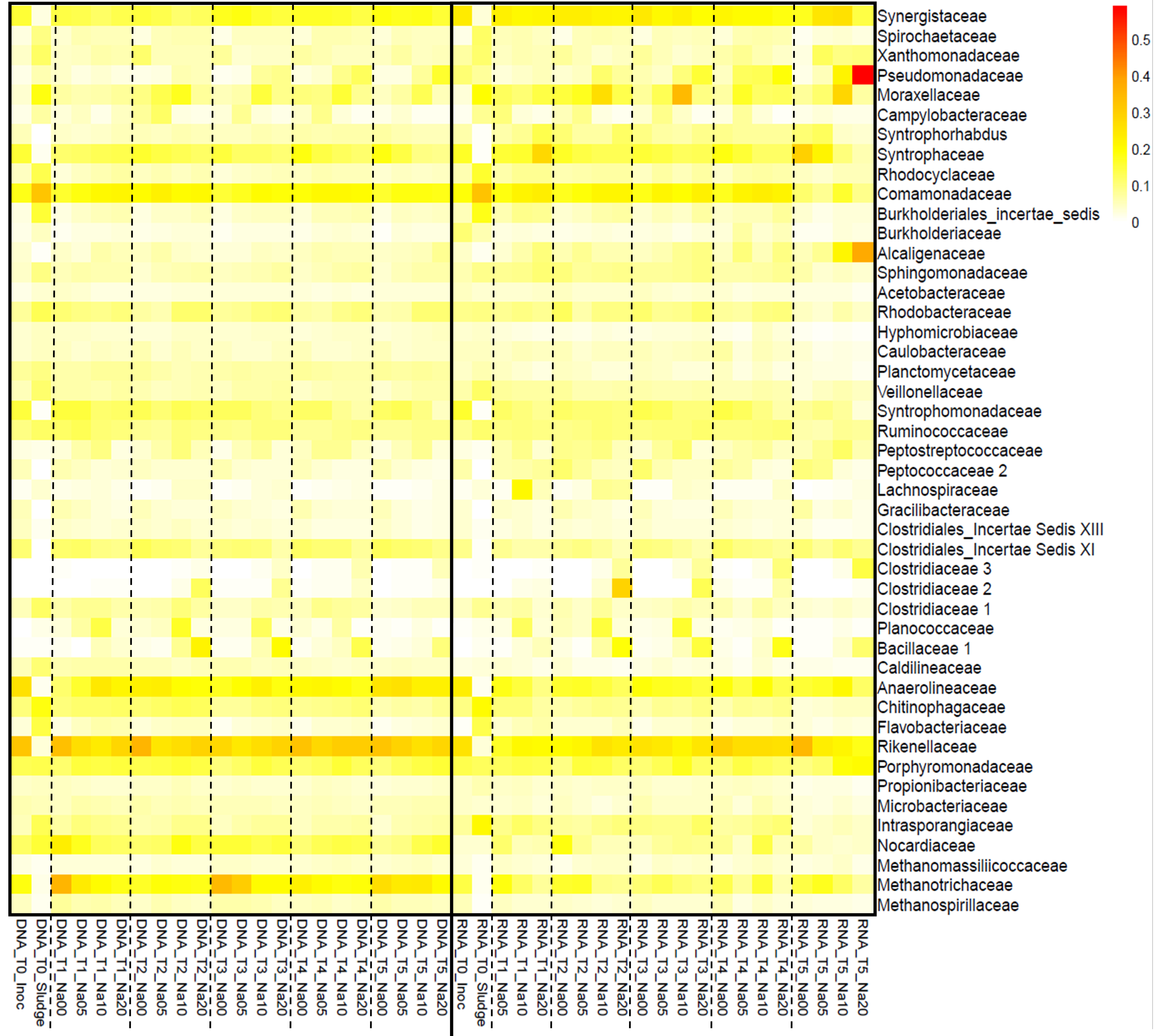
**

**Figure S8** Heatmap representing the microbial community of all samples on family level at a relative abundance > 0.1% averaged over all samples. The colour scale ranges from 0 to 60%. The different samples were labelled according to the time point (T0-T5) and salt concentration (0, 5, 10 and 20 g Na^+^ L^-1^). The inoculum sample (Inoc) at the start of the experiment, as well as the feed sludge (Sludge) were also included in the heatmap.

# S9. Beta diversity analysis


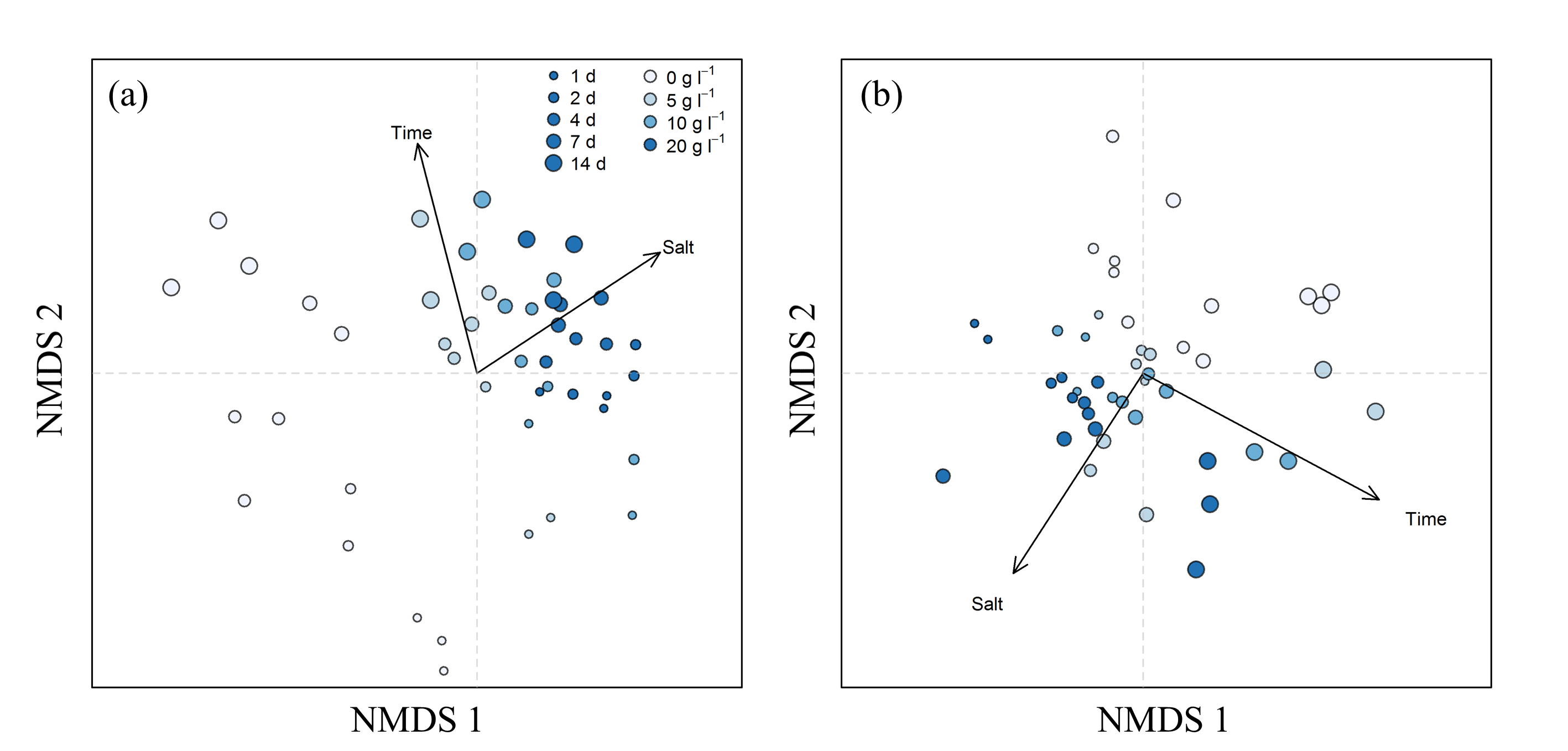


**Figure S9** Non-metric multidimensional scaling analysis of the Bray-Curtis dissimilarity index of the different samples on (a) DNA and (b) RNA level.

# S10. Community variation between DNA and RNA


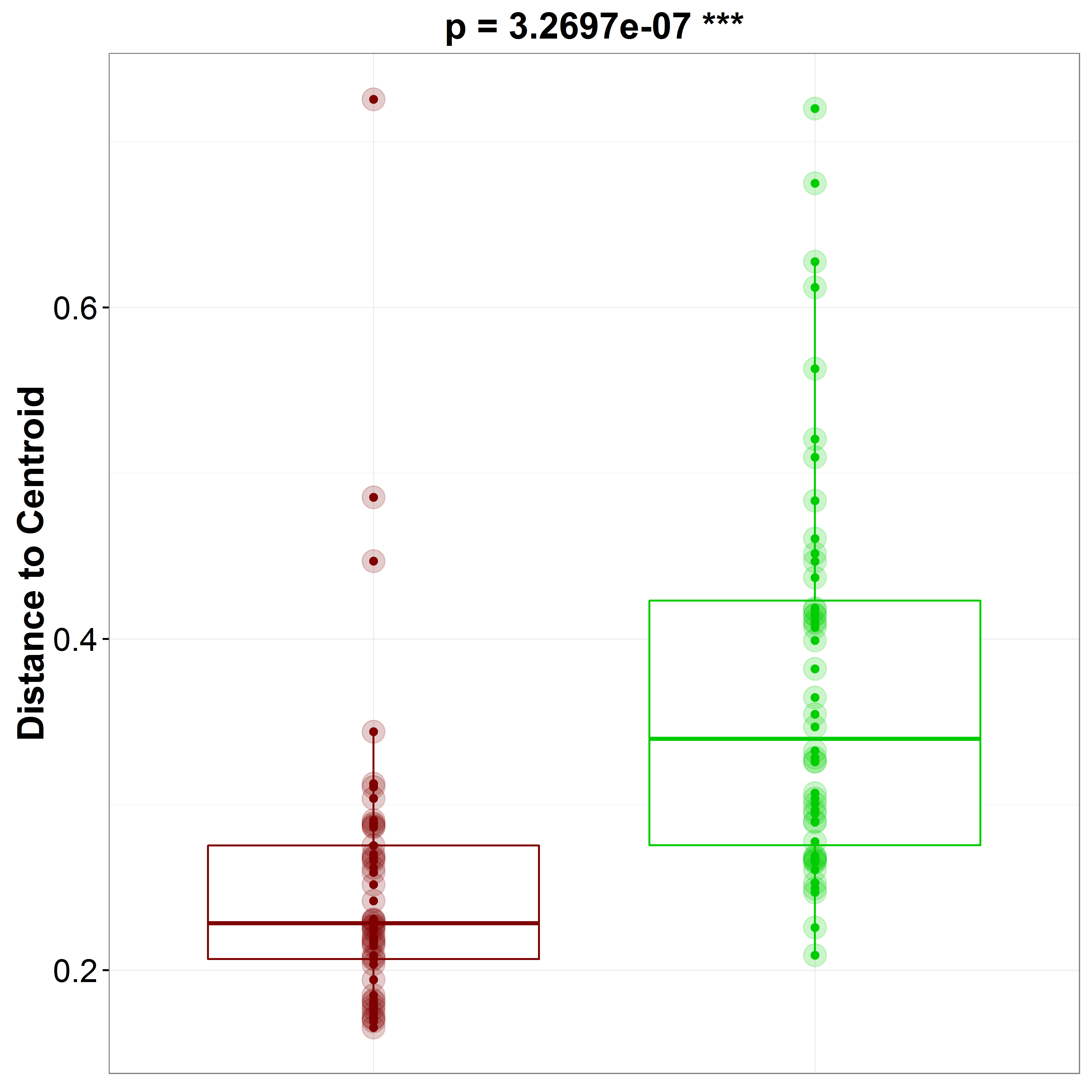


**Figure S10** Overall microbial community variation between the different samples on DNA (red) and RNA (green) level, determined by the distance to the centroid. The distance to the centroid is significantly higher for the RNA samples than the DNA samples (*P* < 0.0001).

# S11. Alpha diversity parameters


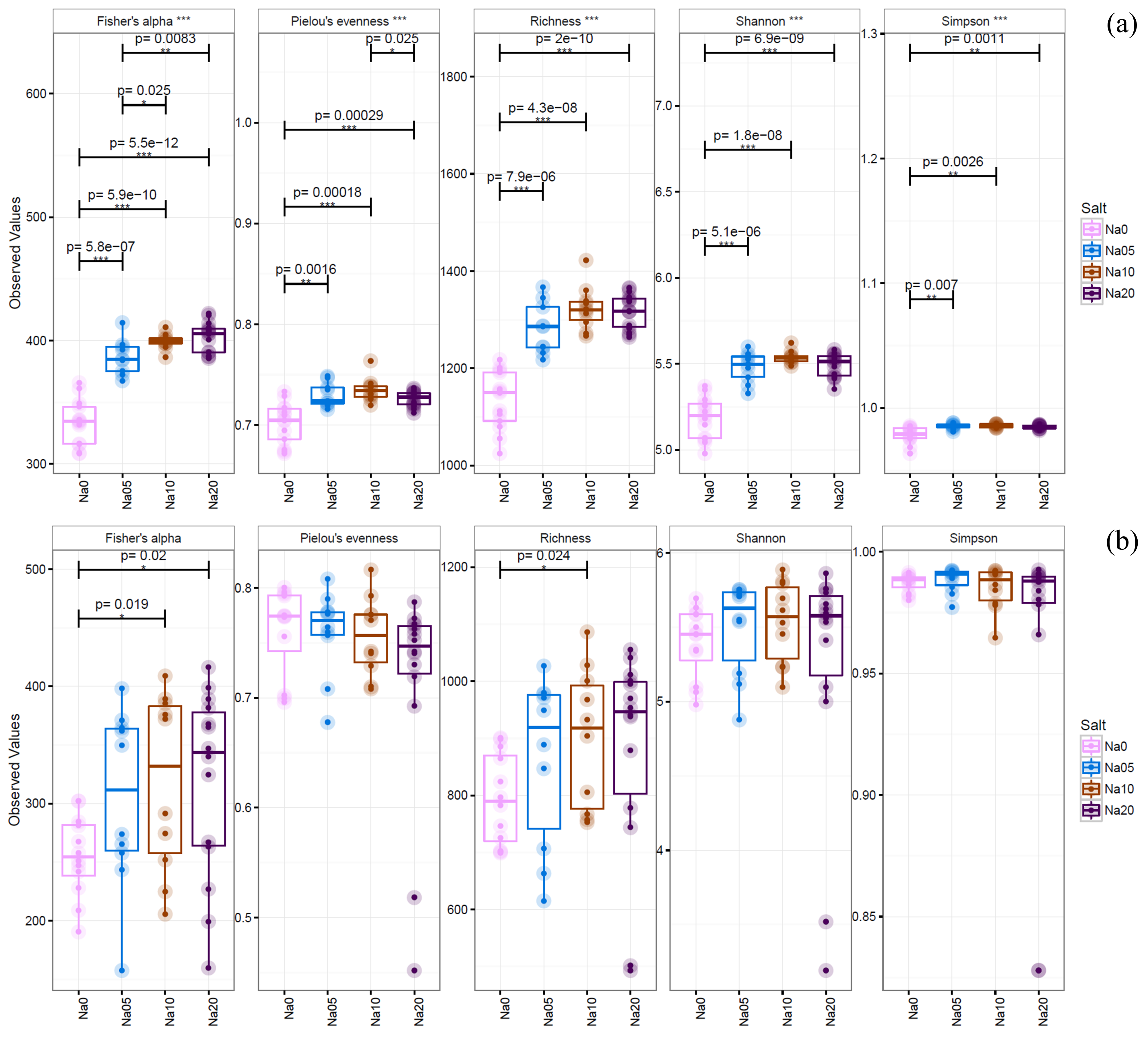


**Figure S11** Boxplots of the alpha diversity metrics on (a) DNA and (b) RNA level for the different salt additions, ranging from 0 (Control) to 20 g Na^+^ L^-1^. Samples were divided in subgroups per salt concentration for comparison of the alpha diversity metrics. A significant difference between any two salt concentrations is marked with * (*P* < 0.05), ** (*P* < 0.01), or *** (*P* < 0.001), based on ANOVA analysis.

**
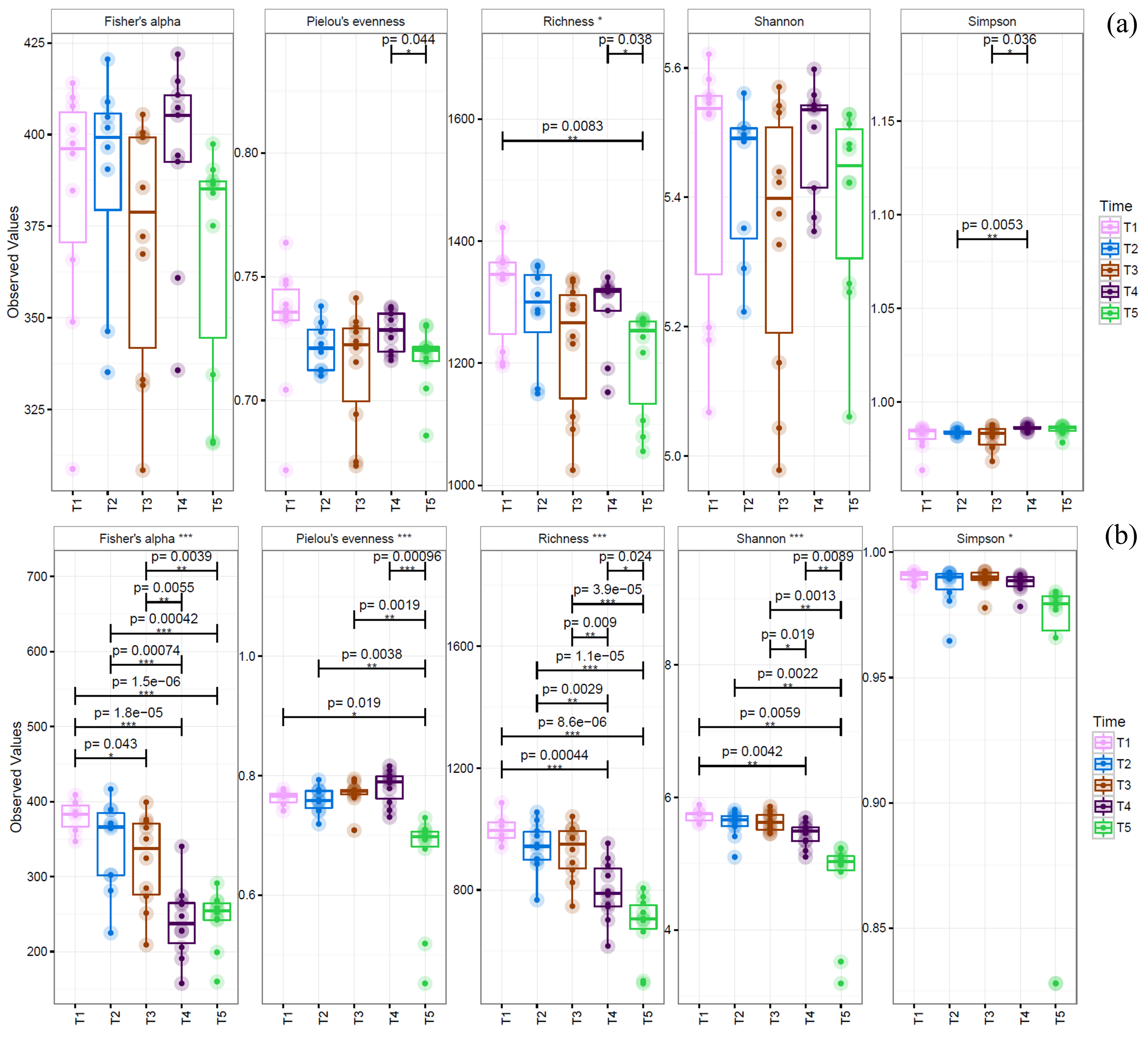
**

**Figure S11** Boxplots of the alpha diversity metrics on (a) DNA and (b) RNA level for the different salt additions, ranging from 0 (Control) to 20 g Na^+^ L^-1^. Samples were divided in subgroups per salt concentration for comparison of the alpha diversity metrics. A significant difference between any two salt concentrations is marked with * (*P* < 0.05), ** (*P* < 0.01), or *** (*P* < 0.001), based on ANOVA analysis.

**Table S4** Overview of the P-values of the multivariate abundance model of the 79 phylotypes that had a relative abundance > 0.1% in at least one sample, and were present in all samples. Adjusted P-values were determined for salt concentration, time and the DNA/RNA ratio, as well as the interaction effect between salt concentration and DNA/RNA ratio and time and DNA/RNA ratio. Highlighted P-values are considered significant at α = 0.05.

|  | P-value | | | | |
| --- | --- | --- | --- | --- | --- |
| Phylotype | Salt | Time | DNA-RNA | Salt*DNA-RNA | Time*DNA-RNA |
| Phy392_Methanobacterium | 0.9926 | 1.0000 | 0.0004 | 1.0000 | 0.9990 |
| Phy577_Methanoculleus | 0.2342 | 0.9654 | 0.1220 | 1.0000 | 0.3583 |
| Phy335_Methanosphaerula | 0.9034 | 1.0000 | 0.8444 | 1.0000 | 0.9990 |
| Phy102_Methanospirillum | 0.1978 | 0.9728 | 0.0002 | 0.9268 | 0.9946 |
| Phy447_Methanospirillum | 0.4807 | 1.0000 | 0.8076 | 0.9996 | 0.9896 |
| Phy54_Methanomicrobiales | 0.0310 | 0.0076 | 0.9750 | 1.0000 | 0.9990 |
| Phy1_Methanosaeta | 0.0244 | 1.0000 | 0.0002 | 0.9268 | 0.9990 |
| Phy127_Methanomassiliicoccus | 0.9996 | 0.9998 | 0.9750 | 0.9996 | 0.5575 |
| Phy332_Archaea | 0.9994 | 1.0000 | 0.0002 | 1.0000 | 0.9990 |
| Phy685_Archaea | 0.0120 | 0.9812 | 0.9750 | 0.9998 | 0.5263 |
| Phy138_Acidimicrobineae | 0.9992 | 0.0002 | 0.0002 | 1.0000 | 0.4071 |
| Phy11_Gordonia | 0.9824 | 0.4219 | 0.0002 | 0.1956 | 0.6115 |
| Phy34_Intrasporangiaceae | 0.0310 | 0.0004 | 0.0834 | 1.0000 | 0.0358 |
| Phy15_Actinomycetales | 0.0310 | 0.9720 | 0.0002 | 1.0000 | 0.0002 |
| Phy27_Actinomycetales | 0.8294 | 0.0052 | 0.0002 | 0.9966 | 0.0002 |
| Phy169_Actinobacteria | 0.9996 | 0.9808 | 0.0002 | 1.0000 | 0.8136 |
| Phy49_Armatimonadetes_gp2 | 0.2158 | 0.0024 | 0.0002 | 0.6963 | 0.0006 |
| Phy63_Armatimonadetes_gp2 | 0.1118 | 0.0464 | 0.0002 | 0.9966 | 0.0006 |
| Phy107_Petrimonas | 0.9034 | 1.0000 | 0.3881 | 1.0000 | 0.9874 |
| Phy35_Porphyromonadaceae | 0.0310 | 0.0008 | 0.8504 | 0.6421 | 0.1904 |
| Phy55_Porphyromonadaceae | 0.0036 | 0.0002 | 0.8904 | 0.1320 | 0.7385 |
| Phy2_Rikenella | 0.9848 | 1.0000 | 0.0002 | 1.0000 | 0.9990 |
| Phy19_Rikenellaceae | 0.9820 | 1.0000 | 0.0006 | 0.9998 | 0.9998 |
| Phy5_Rikenellaceae | 0.0954 | 0.4195 | 0.0374 | 0.9996 | 0.5263 |
| Phy10_Bacteroidetes | 0.0310 | 0.7411 | 0.0004 | 0.9582 | 0.9956 |
| Phy8_Bacteroidetes | 0.0036 | 0.0024 | 0.9622 | 0.9830 | 0.2755 |
| Phy22_Bacteroidetes | 0.0356 | 1.0000 | 0.0056 | 1.0000 | 0.9998 |
| Phy3_Bacteroidetes | 0.0032 | 0.0008 | 0.0002 | 1.0000 | 0.9930 |
| Phy52_Bacteroidetes | 0.0892 | 1.0000 | 0.0048 | 1.0000 | 0.9990 |
| Phy4_Bacteroidetes | 0.9986 | 1.0000 | 0.0702 | 1.0000 | 0.9998 |
| Phy104_Saccharibacteria_genera_incertae_sedis | 0.3613 | 1.0000 | 0.8504 | 0.9998 | 0.2480 |
| Phy86_Anaerolineaceae | 0.7688 | 0.5359 | 0.0232 | 0.4889 | 0.9808 |
| Phy32_Anaerolineaceae | 0.5217 | 0.3083 | 0.0014 | 0.3715 | 0.9896 |
| Phy76_Anaerolineaceae | 0.5143 | 0.5173 | 0.0002 | 0.4353 | 0.9990 |
| Phy37_Anaerolineaceae | 0.9670 | 0.4653 | 0.0002 | 0.7890 | 0.9998 |
| Phy93_Anaerolineaceae | 0.0166 | 0.7844 | 0.0054 | 0.0102 | 0.2202 |
| Phy97_Anaerolineaceae | 0.8802 | 0.3963 | 0.0016 | 0.2743 | 0.8894 |
| Phy78_Anaerolineaceae | 0.8628 | 0.0220 | 0.0006 | 0.6479 | 0.9998 |
| Phy89_Anaerolineaceae | 0.0092 | 0.9232 | 0.9728 | 0.9992 | 0.9634 |
| Phy26_Candidatus_Cloacamonas | 0.0036 | 0.1550 | 0.1386 | 1.0000 | 0.9998 |
| Phy87_Sedimentibacter | 0.0024 | 1.0000 | 0.0050 | 1.0000 | 0.9896 |
| Phy23_Syntrophomonas | 0.0122 | 0.0002 | 0.4245 | 0.9998 | 0.9928 |
| Phy42_Clostridiales | 0.1118 | 0.0002 | 0.0002 | 1.0000 | 0.9990 |
| Phy60_Veillonellaceae | 0.9788 | 0.0026 | 0.9728 | 0.4287 | 0.0088 |
| Phy40_Firmicutes | 0.9986 | 0.0024 | 0.0100 | 0.3251 | 0.0718 |
| Phy30_Firmicutes | 0.0184 | 1.0000 | 0.0002 | 1.0000 | 0.8914 |
| Phy85_Firmicutes | 0.9994 | 1.0000 | 0.3091 | 1.0000 | 0.9998 |
| Phy53_Candidatus_Hydrogenedens | 0.0190 | 1.0000 | 0.0002 | 0.9996 | 0.9990 |
| Phy18_Parcubacteria_genera_ incertae_sedis | 0.0002 | 0.0904 | 0.3775 | 1.0000 | 0.9316 |
| Phy46_Acidovorax | 0.9452 | 0.0008 | 0.8504 | 1.0000 | 0.0002 |
| Phy43_Acidovorax | 0.7688 | 0.0002 | 0.6033 | 1.0000 | 0.0302 |
| Phy69_Brachymonas | 0.6189 | 0.9108 | 0.9750 | 1.0000 | 0.0262 |
| Phy31_Simplicispira | 0.0310 | 0.0024 | 0.7872 | 0.9978 | 0.0042 |
| Phy45_Comamonadaceae | 0.9996 | 0.1210 | 0.6425 | 1.0000 | 0.0002 |
| Phy47_Comamonadaceae | 0.0042 | 0.2298 | 0.8504 | 1.0000 | 0.0082 |
| Phy39_Burkholderiales | 0.9700 | 0.0008 | 0.9478 | 0.9996 | 0.0002 |
| Phy9_Syntrophaceae | 0.0310 | 0.6745 | 0.8838 | 1.0000 | 0.9398 |
| Phy50_Acinetobacter | 0.9996 | 0.0164 | 0.0002 | 1.0000 | 0.9998 |
| Phy75_Thermovirga | 0.0192 | 0.5589 | 0.0050 | 0.9998 | 0.0162 |
| Phy28_Synergistaceae | 0.9928 | 1.0000 | 0.0002 | 1.0000 | 0.8900 |
| Phy61_Synergistaceae | 0.0422 | 1.0000 | 0.9750 | 1.0000 | 0.0176 |
| Phy24_Synergistaceae | 0.0126 | 1.0000 | 0.1220 | 1.0000 | 0.0004 |
| Phy58_Synergistaceae | 0.9806 | 0.0708 | 0.7872 | 1.0000 | 0.0004 |
| Phy96_Synergistaceae | 0.9996 | 0.9232 | 0.1162 | 0.9962 | 0.0036 |
| Phy14_Bacteria | 0.0310 | 0.9956 | 0.8838 | 1.0000 | 0.0164 |
| Phy36_Bacteria | 0.9996 | 1.0000 | 0.9454 | 0.9998 | 0.1218 |
| Phy12_Bacteria | 0.9824 | 1.0000 | 0.0002 | 0.4785 | 0.9866 |
| Phy29_Bacteria | 0.0364 | 0.1210 | 0.0014 | 0.5643 | 0.8890 |
| Phy77_Bacteria | 0.9926 | 1.0000 | 0.0002 | 0.0064 | 0.8370 |
| Phy25_Bacteria | 0.0264 | 1.0000 | 0.8504 | 1.0000 | 0.0038 |
| Phy13_Bacteria | 0.0036 | 1.0000 | 0.9454 | 0.4639 | 0.9264 |
| Phy44_Bacteria | 0.9700 | 1.0000 | 0.0132 | 0.6421 | 0.6603 |
| Phy7_Bacteria | 0.0002 | 0.6745 | 0.0184 | 0.2240 | 0.9928 |
| Phy120_Bacteria | 0.1184 | 0.6417 | 0.2573 | 0.9996 | 0.9896 |
| Phy17_Bacteria | 0.0402 | 1.0000 | 0.0002 | 1.0000 | 0.9998 |
| Phy67_Bacteria | 0.0126 | 0.6745 | 0.9750 | 0.9250 | 0.9990 |
| Phy109_Bacteria | 0.9144 | 0.9586 | 0.0014 | 0.9998 | 0.0004 |
| Phy33_Bacteria | 0.3405 | 0.5865 | 0.8904 | 1.0000 | 0.8136 |
| Phy21_Bacteria | 0.9996 | 0.1308 | 0.0046 | 0.4499 | 0.6991 |

**Table S5** Overview of the model coefficients of the multivariate abundance model of the 79 phylotypes that had a relative abundance > 0.1% in at least one sample, and were present in all samples. Coefficients were determined for salt concentration, time and the DNA/RNA ratio, as well as the interaction effect between salt concentration and DNA/RNA ratio and time and DNA/RNA ratio. A negative value indicates a negative effect of salt or time or a DNA/RNA ratio > 1.

|  | Model coefficient | | | | |
| --- | --- | --- | --- | --- | --- |
| Phylotype | Salt | Time | DNA-RNA | Salt*DNA-RNA | Time*DNA-RNA |
| Phy392_Methanobacterium | -0.010 | 0.014 | -1.126 | -0.012 | 0.033 |
| Phy577_Methanoculleus | 0.034 | 0.055 | 1.427 | 0.028 | -0.156 |
| Phy335_Methanosphaerula | 0.014 | 0.019 | 0.397 | 0.006 | -0.028 |
| Phy102_Methanospirillum | -0.019 | -0.013 | -0.540 | -0.026 | -0.026 |
| Phy447_Methanospirillum | -0.044 | 0.003 | -0.283 | 0.026 | -0.056 |
| Phy54_Methanomicrobiales | -0.034 | 0.058 | 0.022 | 0.006 | -0.020 |
| Phy1_Methanosaeta | -0.040 | 0.006 | -1.015 | -0.023 | -0.015 |
| Phy127_Methanomassiliicoccus | -0.004 | 0.041 | 0.118 | 0.017 | -0.060 |
| Phy332_Archaea | -0.006 | 0.007 | -1.320 | -0.002 | 0.024 |
| Phy685_Archaea | 0.055 | 0.022 | 0.423 | 0.030 | -0.127 |
| Phy138_Acidimicrobineae | -0.004 | -0.097 | 1.388 | -0.011 | -0.068 |
| Phy11_Gordonia | 0.005 | -0.041 | -0.229 | -0.053 | -0.078 |
| Phy34_Intrasporangiaceae | 0.034 | -0.046 | 0.823 | -0.010 | -0.075 |
| Phy15_Actinomycetales | -0.030 | 0.035 | 1.772 | -0.006 | -0.086 |
| Phy27_Actinomycetales | -0.003 | 0.020 | 2.362 | -0.022 | -0.149 |
| Phy169_Actinobacteria | 0.007 | 0.008 | 1.377 | -0.011 | -0.049 |
| Phy49_Armatimonadetes_gp2 | -0.005 | -0.010 | 1.538 | -0.032 | -0.094 |
| Phy63_Armatimonadetes_gp2 | -0.016 | 0.010 | 1.533 | -0.028 | -0.106 |
| Phy107_Petrimonas | -0.027 | 0.000 | -0.883 | 0.021 | 0.046 |
| Phy35_Porphyromonadaceae | 0.009 | 0.040 | -0.901 | 0.024 | 0.077 |
| Phy55_Porphyromonadaceae | 0.062 | -0.089 | 0.458 | -0.036 | -0.042 |
| Phy2_Rikenella | -0.005 | 0.001 | -0.534 | -0.002 | 0.016 |
| Phy19_Rikenellaceae | -0.010 | -0.004 | -0.475 | 0.009 | 0.000 |
| Phy5_Rikenellaceae | -0.010 | 0.006 | -0.449 | -0.008 | 0.041 |
| Phy10_Bacteroidetes | -0.013 | 0.011 | -0.347 | -0.014 | 0.016 |
| Phy8_Bacteroidetes | -0.025 | 0.019 | -0.113 | -0.013 | 0.055 |
| Phy22_Bacteroidetes | -0.015 | -0.004 | -0.224 | -0.003 | -0.003 |
| Phy3_Bacteroidetes | 0.065 | -0.078 | -0.884 | 0.006 | -0.027 |
| Phy52_Bacteroidetes | -0.014 | 0.002 | -0.162 | -0.009 | -0.013 |
| Phy4_Bacteroidetes | -0.001 | 0.009 | -0.198 | -0.006 | -0.003 |
| Phy104_Saccharibacteria_genera_ incertae_sedis | 0.021 | 0.022 | 0.289 | -0.011 | -0.057 |
| Phy86_Anaerolineaceae | 0.001 | 0.041 | 0.112 | -0.034 | -0.030 |
| Phy32_Anaerolineaceae | -0.003 | 0.040 | 0.017 | -0.033 | -0.024 |
| Phy76_Anaerolineaceae | -0.005 | 0.032 | -0.218 | -0.028 | -0.015 |
| Phy37_Anaerolineaceae | -0.003 | 0.032 | -0.699 | -0.021 | 0.001 |
| Phy93_Anaerolineaceae | 0.053 | -0.002 | 0.376 | -0.051 | -0.057 |
| Phy97_Anaerolineaceae | 0.002 | 0.038 | 0.075 | -0.032 | -0.031 |
| Phy78_Anaerolineaceae | 0.000 | 0.051 | -0.171 | -0.030 | -0.009 |
| Phy89_Anaerolineaceae | -0.022 | 0.036 | 0.459 | -0.020 | -0.034 |
| Phy26_Candidatus_Cloacamonas | -0.034 | 0.050 | 0.478 | -0.005 | -0.013 |
| Phy87_Sedimentibacter | -0.056 | -0.016 | -0.683 | -0.013 | 0.037 |
| Phy23_Syntrophomonas | -0.045 | -0.087 | 0.055 | -0.014 | -0.024 |
| Phy42_Clostridiales | -0.028 | -0.087 | -0.679 | -0.002 | 0.016 |
| Phy60_Veillonellaceae | 0.021 | -0.015 | 0.705 | -0.027 | -0.068 |
| Phy40_Firmicutes | -0.022 | 0.012 | -0.270 | 0.031 | 0.056 |
| Phy30_Firmicutes | -0.027 | 0.016 | -0.294 | -0.006 | -0.029 |
| Phy85_Firmicutes | -0.007 | -0.003 | -0.368 | 0.008 | 0.008 |
| Phy53_Candidatus_Hydrogenedens | -0.033 | 0.035 | 1.494 | -0.028 | -0.031 |
| Phy18_Parcubacteria_genera_ incertae_sedis | -0.067 | -0.028 | -0.132 | 0.010 | -0.044 |
| Phy46_Acidovorax | 0.006 | -0.019 | 0.433 | 0.001 | -0.105 |
| Phy43_Acidovorax | 0.011 | -0.062 | 0.244 | -0.002 | -0.077 |
| Phy69_Brachymonas | -0.014 | 0.013 | 0.430 | 0.000 | -0.081 |
| Phy31_Simplicispira | 0.019 | -0.022 | 0.215 | 0.011 | -0.086 |
| Phy45_Comamonadaceae | 0.000 | 0.004 | 0.415 | 0.003 | -0.114 |
| Phy47_Comamonadaceae | 0.036 | 0.000 | 0.342 | 0.001 | -0.092 |
| Phy39_Burkholderiales | 0.001 | -0.014 | 0.427 | 0.009 | -0.110 |
| Phy9_Syntrophaceae | -0.033 | -0.006 | 0.382 | 0.000 | -0.038 |
| Phy50_Acinetobacter | -0.004 | -0.071 | 0.996 | -0.013 | -0.002 |
| Phy75_Thermovirga | -0.023 | 0.011 | 0.924 | -0.011 | -0.075 |
| Phy28_Synergistaceae | -0.018 | 0.044 | 2.076 | 0.007 | -0.047 |
| Phy61_Synergistaceae | -0.025 | 0.026 | 0.491 | 0.002 | -0.080 |
| Phy24_Synergistaceae | -0.025 | 0.038 | 0.663 | 0.001 | -0.076 |
| Phy58_Synergistaceae | 0.007 | 0.008 | 0.574 | -0.002 | -0.073 |
| Phy96_Synergistaceae | -0.009 | 0.032 | 0.778 | 0.014 | -0.096 |
| Phy14_Bacteria | 0.030 | 0.028 | 0.656 | 0.004 | -0.093 |
| Phy36_Bacteria | 0.006 | 0.018 | 0.372 | -0.014 | -0.059 |
| Phy12_Bacteria | 0.017 | 0.020 | -0.130 | -0.029 | -0.025 |
| Phy29_Bacteria | -0.015 | 0.052 | 0.048 | -0.031 | -0.038 |
| Phy77_Bacteria | 0.009 | 0.015 | 0.009 | -0.053 | -0.040 |
| Phy25_Bacteria | -0.028 | 0.032 | 0.441 | -0.006 | -0.098 |
| Phy13_Bacteria | 0.043 | 0.014 | 0.348 | -0.028 | -0.031 |
| Phy44_Bacteria | 0.002 | 0.027 | 0.156 | -0.030 | -0.046 |
| Phy7_Bacteria | -0.028 | 0.031 | 0.086 | -0.030 | -0.021 |
| Phy120_Bacteria | -0.026 | 0.042 | 0.361 | 0.012 | -0.029 |
| Phy17_Bacteria | -0.024 | 0.007 | -0.573 | 0.006 | 0.002 |
| Phy67_Bacteria | -0.036 | 0.030 | -0.130 | 0.020 | -0.016 |
| Phy109_Bacteria | -0.007 | 0.016 | 0.247 | -0.016 | -0.115 |
| Phy33_Bacteria | -0.011 | 0.037 | 0.332 | -0.001 | -0.035 |
| Phy21_Bacteria | 0.009 | -0.019 | 0.110 | -0.028 | -0.039 |
